# A method for estimation of elasticities in metabolic networks using steady state and dynamic metabolomics data and linlog kinetics

**DOI:** 10.1186/1471-2105-7-540

**Published:** 2006-12-21

**Authors:** I Emrah Nikerel, Wouter A van Winden, Walter M van Gulik, Joseph J Heijnen

**Affiliations:** 1 Department of Biotechnology, TU Delft, Julianalaan 67, 2628 BC Delft, The Netherlands

## Abstract

**Background:**

Dynamic modeling of metabolic reaction networks under *in vivo *conditions is a crucial step in order to obtain a better understanding of the (dis)functioning of living cells. So far dynamic metabolic models generally have been based on mechanistic rate equations which often contain so many parameters that their identifiability from experimental data forms a serious problem. Recently, approximative rate equations, based on the linear logarithmic (linlog) format have been proposed as a suitable alternative with fewer parameters.

**Results:**

In this paper we present a method for estimation of the kinetic model parameters, which are equal to the elasticities defined in Metabolic Control Analysis, from metabolite data obtained from dynamic as well as steady state perturbations, using the linlog kinetic format. Additionally, we address the question of parameter identifiability from dynamic perturbation data in the presence of noise. The method is illustrated using metabolite data generated with a dynamic model of the glycolytic pathway of *Saccharomyces cerevisiae *based on mechanistic rate equations. Elasticities are estimated from the generated data, which define the complete linlog kinetic model of the glycolysis. The effect of data noise on the accuracy of the estimated elasticities is presented. Finally, identifiable subset of parameters is determined using information on the standard deviations of the estimated elasticities through Monte Carlo (MC) simulations.

**Conclusion:**

The parameter estimation within the linlog kinetic framework as presented here allows the determination of the elasticities directly from experimental data from typical dynamic and/or steady state experiments. These elasticities allow the reconstruction of the full kinetic model of *Saccharomyces cerevisiae*, and the determination of the control coefficients. MC simulations revealed that certain elasticities are potentially unidentifiable from dynamic data only. Addition of steady state perturbation of enzyme activities solved this problem.

## Background

Metabolic activities of living cells are accomplished by a well regulated, highly coupled network of numerous enzyme catalyzed reactions and selective membrane transport systems. To engineer such systems, enzymatic, transport and regulatory functions of the cells are manipulated via the use of recombinant DNA technology [1]. Within the purpose of metabolic engineering, i.e. rational redesign of the metabolic systems, the highly relevant question of which (combination of) perturbation should be applied in order to increase the productivity of the microorganism is addressed. The answer to this question requires information on both the regulatory level and the metabolic level. Ter Kuile and Westerhoff showed by their Hierarchical Control Analysis that the pathway flux is rarely controlled solely by gene expression, but that metabolite levels are also relevant [2].

In this context, we focus on the metabolic reaction network level. Modeling metabolic reaction systems is usually based on stoichiometric sometimes followed by kinetic modeling.

One of the initial steps in the modeling of metabolic reaction networks is to determine the structure and steady state characteristics of a given network using stoichiometric information alone. Steady state models describe time invariant fluxes, gathered from steady state experiments; hence they reflect the structural characteristics of the system. Metabolic Flux Analysis (MFA) and Metabolic Network Analysis (MNA) were developed as powerful tools to analyze such flux data. At steady state, the mass balances over the metabolites in the metabolic network yield a set of linear relations between the metabolic fluxes which can be expressed as:

**S·v **= **0 **    (1)

Where **S **is the (*m *× *r*) stoichiometric matrix and **v **is the (*r *× 1) vector of metabolic fluxes, where *m *is the number of balanced metabolites and *r *is the number of fluxes. Here, the system is in most of the cases underdetermined so that there are an infinite number of possible solutions. The realized solution depends on the kinetic properties of the involved reactions; this information is seldom known. To bypass the need of information on kinetics of individual reactions, alternative mathematical approaches have been proposed in the past to obtain a unique solution. An example is the constraint based optimization approach which is based on assumed optimality criteria, e.g. maximum growth, given biochemical, thermodynamic and irreversibility constraints and maximal reaction rates [3-5]. Later, Segre *et al*. proposed the optimality constraint that requires maximization of biomass formation while minimization of metabolic adjustment (MOMA) in order to obtain a unique flux distribution of a mutant strain. In their approach, they defend their optimality criterion that a knock-out mutant strain would optimize its biomass production rate by changing minimally its metabolic fluxes from the wild type strain [6]. An alternative metabolic modeling framework which uses a fitness function is the cybernetic approach. This approach assumes that an organism is an optimal strategist in utilizing all available sources with maximum efficiency. The expression and activity of the enzymes that catalyze network functionality are regulated by cybernetic control variables obtained from the solution of a constrained optimization problem [7-9].

Despite a number of successful applications especially in mixed substrate and prediction of knock-out lethality, all stoichiometric modeling approaches have their limitations, e.g. they can not predict time courses of the cellular processes, are based on "assumed goals" of the cell, and do not give insight in molecular events occurring in the cells, since information about the kinetic properties of the individual enzymes are not required. In the light of the above arguments, it is apparent that, to advance our understanding of the (dis)functioning of living cells a systems biology approach is needed, whereby the use of dynamic mathematical models of metabolic reaction networks to describe the complex kinetic behavior and interactions (allosterical, feedback and feed forward effects, cofactor coupling, compartmentation, intracellular transport, etc.) is becoming increasingly relevant.

The kinetic behavior of many important enzymes occurring in metabolic networks have been studied extensively, however these studies have generally been performed under non-physiological conditions in test tubes (*in vitro*), and therefore the applicability of these results to the *in vivo *metabolism is doubtful [10-13]. Teusink *et al*. showed that discrepancies exist between the *in vivo *measured changes of the concentrations of the glycolytic metabolites and their estimates using models based on mechanistic rate equations and *in vitro *determined parameters [12]. This basic problem invalidates detailed models of metabolism containing kinetic parameters which have been determined *in vitro*. Therefore, it is preferred to base the kinetic analysis of metabolic networks on *in vivo *studies of intact cellular networks. These *in vivo *studies are based on steady state and/or dynamic perturbations of a metabolic network starting from a reference steady state that is defined by its fluxes, enzyme activities, metabolite levels, extracellular concentrations, and kinetic parameters.

It is also important to notice that the number of parameters that is typically involved in the traditional mechanistic equations is very large which causes identifiability problems due to parameter insensitivity. Despite the information richness of data obtained from dynamic perturbations in *in vivo *experiments, there is a limit for identification of the parameters. For these reasons, it seems justified not to limit ourselves to the available complex mechanistic enzyme kinetics which must also be considered as approximations of the true *in vivo *behavior [14,15]. Approximative enzyme kinetic formats, which contain much fewer kinetic parameters are therefore of general interest. An overview of different approximative kinetic formats (linear, power law, loglin and linlog kinetics) used in metabolic network modeling is given by Heijnen [16].

One of the proposed formats is, linlog kinetics, which has been introduced for modeling of *in vivo *kinetics and for metabolic redesign, and shown to have a good approximation quality, standardized format and relatively few parameters [16,17]. In linlog kinetics, all the rate equations have the same mathematical structure in which the relation between rates and enzyme levels is proportional, while for metabolite levels, a linear sum of logarithmic concentration terms is proposed. All variables are considered relative to a reference steady state (Eq. (2)). The linlog approximation is valid in the neighborhood of the reference state (defined by *J*^0^, *x*^0 ^and *c*^0 ^in equation (2)), but quite large changes of metabolite concentrations, enzyme levels and fluxes are allowed [17]. The parameters (εxv
 MathType@MTEF@5@5@+=feaafiart1ev1aaatCvAUfKttLearuWrP9MDH5MBPbIqV92AaeXatLxBI9gBaebbnrfifHhDYfgasaacH8akY=wiFfYdH8Gipec8Eeeu0xXdbba9frFj0=OqFfea0dXdd9vqai=hGuQ8kuc9pgc9s8qqaq=dirpe0xb9q8qiLsFr0=vr0=vr0dc8meaabaqaciaacaGaaeqabaqabeGadaaakeaaiiGacqWF1oqzdaqhaaWcbaGaemiEaGhabaGaemODayhaaaaa@3175@ and εcv
 MathType@MTEF@5@5@+=feaafiart1ev1aaatCvAUfKttLearuWrP9MDH5MBPbIqV92AaeXatLxBI9gBaebbnrfifHhDYfgasaacH8akY=wiFfYdH8Gipec8Eeeu0xXdbba9frFj0=OqFfea0dXdd9vqai=hGuQ8kuc9pgc9s8qqaq=dirpe0xb9q8qiLsFr0=vr0=vr0dc8meaabaqaciaacaGaaeqabaqabeGadaaakeaaiiGacqWF1oqzdaqhaaWcbaGaem4yamgabaGaemODayhaaaaa@314B@) in the kinetic equations are the same scaled elasticities (εxv=x0J0∂v∂x|x=x0)
 MathType@MTEF@5@5@+=feaafiart1ev1aaatCvAUfKttLearuWrP9MDH5MBPbIqV92AaeXatLxBI9gBaebbnrfifHhDYfgasaacH8akY=wiFfYdH8Gipec8Eeeu0xXdbba9frFj0=OqFfea0dXdd9vqai=hGuQ8kuc9pgc9s8qqaq=dirpe0xb9q8qiLsFr0=vr0=vr0dc8meaabaqaciaacaGaaeqabaqabeGadaaakeaadaqadaqaaGGaciab=v7aLnaaDaaaleaacqWG4baEaeaacqWG2bGDaaGccqGH9aqpdaWcaaqaaiabdIha4naaCaaaleqabaGaeGimaadaaaGcbaGaemOsaO0aaWbaaSqabeaacqaIWaamaaaaaOWaaqGaaeaadaWcaaqaaiabgkGi2kabdAha2bqaaiabgkGi2kabdIha4baaaiaawIa7amaaBaaaleaacqWG4baEcqGH9aqpcqWG4baEdaahaaadbeqaaiabicdaWaaaaSqabaaakiaawIcacaGLPaaaaaa@45B3@ that are used in Metabolic Control Analysis (MCA). It is important to note that the elasticity parameters appear in the model in a linear fashion.

vJ0=ee0(1+εxvln⁡(xx0)+εcvln⁡(cc0))     (2)
 MathType@MTEF@5@5@+=feaafiart1ev1aaatCvAUfKttLearuWrP9MDH5MBPbIqV92AaeXatLxBI9gBaebbnrfifHhDYfgasaacH8akY=wiFfYdH8Gipec8Eeeu0xXdbba9frFj0=OqFfea0dXdd9vqai=hGuQ8kuc9pgc9s8qqaq=dirpe0xb9q8qiLsFr0=vr0=vr0dc8meaabaqaciaacaGaaeqabaqabeGadaaakeaadaWcaaqaaiabdAha2bqaaiabdQeaknaaCaaaleqabaGaeGimaadaaaaakiabg2da9maalaaabaGaemyzaugabaGaemyzau2aaWbaaSqabeaacqaIWaamaaaaaOWaaeWaaeaacqaIXaqmcqGHRaWkiiGacqWF1oqzdaqhaaWcbaGaemiEaGhabaGaemODayhaaOGagiiBaWMaeiOBa42aaeWaaeaadaWcaaqaaiabdIha4bqaaiabdIha4naaCaaaleqabaGaeGimaadaaaaaaOGaayjkaiaawMcaaiabgUcaRiab=v7aLnaaDaaaleaacqWGJbWyaeaacqWG2bGDaaGccyGGSbaBcqGGUbGBdaqadaqaamaalaaabaGaem4yamgabaGaem4yam2aaWbaaSqabeaacqaIWaamaaaaaaGccaGLOaGaayzkaaaacaGLOaGaayzkaaGaaCzcaiaaxMaadaqadaqaaiabikdaYaGaayjkaiaawMcaaaaa@5758@

When the elasticities are known, a full dynamic model of the whole metabolic network can be set up using linlog kinetics. Such a model allows in principle the calculation of control coefficients also under dynamic conditions. In linlog kinetics, the elasticities are the kinetic parameters represented in the elasticity matrix. From these, and a given network structure specified in the form of a stoichiometric matrix, the control coefficients for a reference condition can be calculated from the summation and connectivity relations developed in the framework of MCA. Also the change in control coefficients upon large changes in enzyme levels can be calculated [18]. Moreover, the linlog formulation enables the analytical solution of the mass balances for steady state metabolite and flux levels in the metabolic network, providing the solution of the metabolic redesign problem, i.e. determination of the optimal enzyme levels that maximize a certain flux while the total amount of enzyme and the metabolite levels are constrained. Visser *et al*., reported a successful application of linlog kinetics in an *in silico *study that aims to solve this metabolic redesign problem [19].

In order to determine the kinetic parameters of a model of a given *in vivo *metabolic system, *in vivo *perturbations of the complete metabolic network have to be performed. There are two main types of perturbations that can be imposed on the system: steady state and dynamic perturbations. In steady state perturbations, usually the enzyme activity of one (sometimes more) of the reactions is changed by adding specific inhibitors or activators or by genetically changing the enzyme activity, resulting in a new steady state. In steady state perturbations an important problem has been addressed by Kacser and Burns, which is the determination of the set of reactions that has to be perturbed in steady state fashion, in order to be able to determine all elasticities for a given metabolic network which resulted in their "double modulation" [20]. They showed that for a simple linear chain of reaction, perturbation of the activities of the first and last enzymes allows determination of the elasticities of all enzymes of the chain under the condition that each enzyme is only responsive to its substrate and product, which rules out the fact that feedback loops are present. The theoretical basis is presented in later studies which showed that determination of the elasticities for any enzyme in such a simple chain requires two perturbations, one upstream and one downstream of the enzyme concerned [21]. Giersch and Cornish-Bowden extended the double perturbation approach to more complex pathways containing branch points, regulatory loops, and conserved moieties and they provided guidelines to list the possible reactions to be modulated in order to determine the elasticities for arbitrary metabolic networks [22]. From the obtained list a minimum set of steady state perturbations, to be imposed on a specific network, can be chosen. As an alternative to the analysis of Giersch and Cornish-Bowden, Hofmeyr and Cornish-Bowden offered co-response analysis, to identify the mono-functional units that respond together to any perturbation applied [23]. These mono-functional units have to be dissected in order to determine the elasticities belonging to these groups.

Linlog kinetics has been successfully applied to estimate the elasticity parameters for a linear pathway from sets of steady state metabolite concentrations and enzyme activities [18]. Additionally, Heijnen *et al*. proposed a method to obtain flux control coefficients around a branch point, from large enzyme perturbation leading to large steady state flux perturbations, using the linlog kinetic format [24]. This approach allows obtaining explicit solutions for steady state flux and metabolite levels as a function of large changes in enzyme activities.

An alternative to steady state perturbations are dynamic perturbations, in which the system, being initially at the reference state, is disturbed to create time dependent data of transient metabolite levels. The dynamic metabolite profiles are typically obtained as a series of snapshots in time. A recent example of these are *in vivo *measurements of a number of metabolites in rather dense time sequences of a few seconds or minutes, using 'rapid sampling' methods with subsequent high precision metabolite measurement techniques [25-28]. Transient data are rich in information and allow determination of the time hierarchy of the different elements of the metabolic network and the causal relationships between the network elements. It is possible to exploit these data to estimate the parameters of a traditional full kinetic model [13,29]. Such a model can subsequently be used to calculate the values of the elasticities at a given reference steady state. Alternatively, when the linlog kinetic format is used the elasticities can be directly estimated from the transient data. This was demonstrated by Kresnowati *et al*. who estimated the elasticities of a small example network from transient concentration data, assuming that the dynamic fluxes are unknown [30].

An important difference between steady state and dynamic perturbations is that in the former both the steady state enzyme levels (*e*_*i*_) and the metabolite concentrations (*x*_*i*_) have to be measured, whereas in rapid dynamic perturbation experiments only *x*_*i *_is required because the enzyme activities can be considered constant within a sufficiently short time window following the perturbation. Note that flux data are required in both methods. However, fluxes are not independent variables as they follow from the measured intra- and extracellular metabolite concentrations and the proper mass balances.

In this work, we present a method to estimate kinetic parameters (elasticities), using linlog kinetics, using metabolome data obtained from steady state and dynamic perturbation experiments. As such, this can be considered as an extension of the work by Visser and Heijnen [17], Wu *et al*., [18] and Kresnowati *et al*., [30] who have provided the theoretical framework and a small practical application of the linlog kinetic format. We first generalize the notation to make it applicable to networks of arbitrary size and complexity. Additionally, we address further the question of parameter identification from experimental data. We monitor the propagation of error throughout the proposed parameter estimation procedure and we determine the subset of identifiable parameters. To illustrate the proof of principle, we apply the presented theory on *in silico *generated data, with realistic experimental settings, to be able to compare the obtained results with the "known truth". This data is generated using a previously published glycolytic pathway model [31] of *Saccharomyces cerevisiae*.

## Results

### In silico pulse experiments

**Figure 1:**
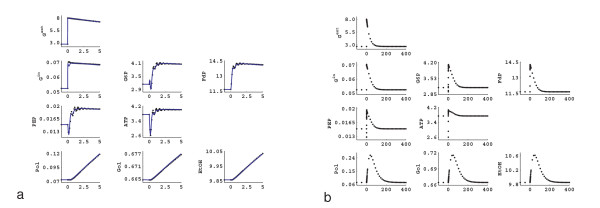
Response of the network to an increase in external glucose concentration. a) First five minutes, a "rapid sampling" experiment (black) and the simulation of the same perturbation with linlog kinetics (blue) using the elasticities estimated from the experimental data from dynamic perturbation. b) Long term response of the cells to the glucose pulse. The time is presented in minutes; intracellular metabolites are given in *μ*mol gDW^-1^; extracellular metabolites are given in mM.

In these experiments, the steady state was perturbed by increasing the extracellular glucose concentration. When the external glucose concentration is increased, the intracellular glucose concentration also increases as the sugar is transported through facilitated diffusion. From Figure 1a it can be seen that the G6P concentration increases around 6% in the first five seconds and then starts to decrease, as the ATP concentration drops due to the phosphorylation of the glucose in the hexokinase reaction. ATP starts to recover after about 15 seconds due to increased increase ATP production downstream of glycolysis. This is in agreement with the literature on the glucose uptake dynamics following a sudden increase in the external sugar concentration [32]. Following this ATP fluctuation, the PEP concentration also decreases and then recovers. In the first five minutes, the concentrations of the external metabolites increase because of their increased production.

After this initial period of 2 min, in which the external glucose concentration is nearly constant, the concentration of external glucose slowly decreases due to continuous consumption and wash-out from the reactor. The cells follow this glucose drop by dropping the levels of intracellular metabolites. For the extracellular products (polysaccharides, glycerol, ethanol) the washout from the reactor is larger than their production and hence their concentration also drops. After about 200 minutes, the original steady state is restored (Figure 1b). Such a pulse experiment therefore always delivers a highly dynamic dataset, followed by a pseudo steady state dataset.

### Parameter estimation

**Figure 2:**
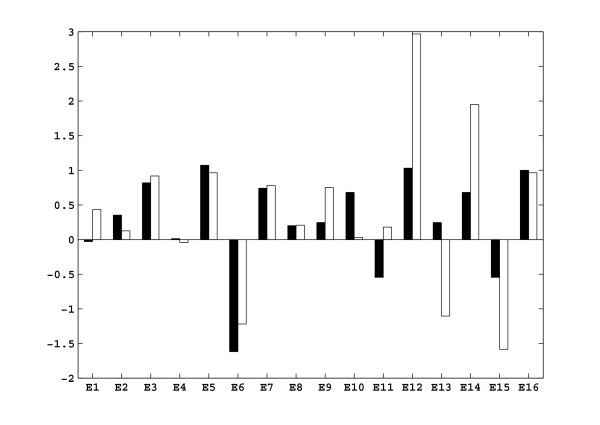
Results of the estimation of the elasticities using dynamic data only, comparison of the theoretical elasticities (black), with the linlog elasticities (white).

The dynamic data obtained during the first five minutes of the glucose pulse experiment (Figure 1a) were used to estimate the elasticities via the linlog parameter estimation procedure outlined in the Methods section (see section Determining elasticities from dynamic perturbation data). In Figure 1a, the simulation of the same dynamic perturbation using the estimated elasticities is also given. In Figure 2, the estimated elasticities are compared with the theoretically calculated elasticities, derived from mechanistic rate equations at the reference state.

Although the linlog simulation results do not differ much from the noise-free experimental data generated with the mechanistic model, some estimated elasticities are far from the expected theoretical values. The difference is in some cases in magnitude, for other cases there is a sign contradiction. Specifically, 6 out of 16 elasticities (E3, E5, E6, E7, E8, E16) are predicted in very good agreement with the theoretical ones (difference being around 10%), 6 estimated elasticities (E2, E9, E10, E12, E14, E15) agree in sign, but the magnitudes differ by more than 50% (in some cases up to 200%), and 4 elasticities (E1, E4, E11, E13) are estimated with a sign contradiction. Notice that in the case of E12, the 200% deviation is already expected. This elasticity explains the effect of G6P on the polysaccharide formation rate and in the mechanistic model this effect is explained with a Hill type kinetic equation with a Hill coefficient of 8.25, hence this rate is very sensitive to the changes in G6P. To mimic this behavior, E12 is estimated to be much higher than its theoretical value.

**Figure 3:**
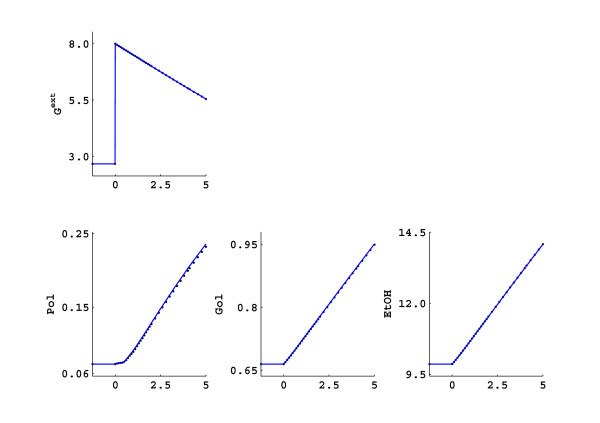
The results of the new experimental design (The inlet and outlet of the reactor is blocked just after the glucose is increased. The biomass concentration is 15 gDW L^-1^, see text for further details in the experimental design) (black) and simulation of the same perturbation with linlog kinetics (blue).

The poor identification of the elasticities has two possible reasons: either the experimental design is poor or there is a structural problem resulting that some interactions in the network can not be resolved from the available information. From an experimentalist point of view, it can be seen from Figure 1a that the changes in the intracellular metabolites during the first 300 seconds are between 25–30% which can be easily detected with the current measurement techniques [33]. The extracellular glucose and polysaccharides also change to a detectable extent, but the changes in the ethanol (and therefore glycerol) are only 2–3% which is hard to detect. To check if the poor experimental design causes problems in the parameter identification, we have altered the experimental design to create larger changes in these concentrations. First the biomass concentration was increased to 15 gDW L^-1^. Also at the same moment that the fermentor was pulsed with the glucose, the inflow and outflow of the fermentor was stopped so the operation was effectively switched from though-flow mode to batch mode, and the extracellular metabolites were not washed out anymore. This allowed more rapid accumulation of the secreted products resulting in much larger changes. Also, the change in the extracellular glucose concentration was more pronounced because there was no further addition of glucose after the pulse. These changes did not result in considerable changes in the intracellular metabolite profiles; they follow the slight change in the external glucose profile. The results of the new experimental design are depicted in Figure 3 which represents the new experimental data where only the significantly changed external metabolite profiles are depicted, together with the linlog simulation using the estimated elasticities. However, no significant improvement was achieved in the identification of the elasticities compared to the initial experimental design (data not shown). Hence it can be concluded that the problem is not due to poor experimental design.

A closer look at the estimated elasticities reveals that the elasticities belonging to the part of the metabolic network, between V_HK _and V_GAPD _are correctly estimated, whereas the estimation of the elasticities belonging to the lower part of glycolysis and the uptake reaction is poor. This is mainly due to the fact that the information content of the metabolite data is insufficient to resolve the complex interactions of metabolites and enzymes. Specifically, in the V_PK _reaction rate, the feed forward activation of FdP and the mass action effect of PEP are assumed. The perturbation of the system via an increase in the external glucose concentration results in an increase in the V_PK _flux. However, this does not allow a separate determination as to what extent the change in V_PK _is due to mass action effect of PEP or due to feed forward effect of FdP. This results in identifiability problems for the elasticity parameters that describe the ethanol production (E9, E10, and E11). The same argument is valid for the elasticities belonging to the glycerol producing reaction, since in the *in silico *network the glycerol production is assumed to be proportional with the ethanol production; hence the same combined effect is seen in the glycerol production, resulting in an identifiability problem for the latter triplet of elasticities (E13, E14, E15)

In order to analyze the information content of the data, the Fisher Information Matrix (FIM) is calculated as described in [30]. FIM is an indicator of the information content with respect to the parameters and due to the parameter linearity in linlog kinetics FIM is independent of the values of the kinetic parameters. It can be calculated as **Y**^T^**Y**, **Y **being the design matrix appearing in equation (9). The singular values of the FIM hold information on the number of linear dependencies between the columns of the data matrix. In our system, we checked the singular values of FIM and concluded that not all the singular values are equally significant, which shows that there are some colinearities within the data matrix. This is also evident from the condition number, which equals the ratio of the highest to the lowest eigenvalues of the matrix and is ideally 1 in a completely uncorrelated case. In this case the condition number was calculated to be 5.0 × 10^6^. This fact supports the previous argument that using only one single set of dynamic data is not sufficient to resolve all the elasticity values.

**Figure 4:**
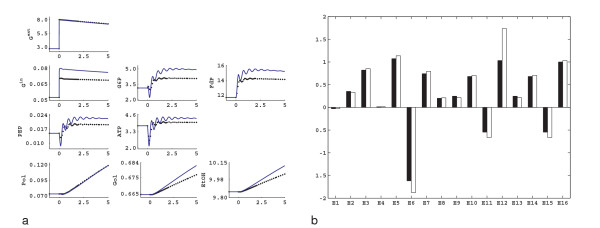
Linlog model simulation of the dynamic perturbation of Figure 1a using elasticities obtained from steady state data only. a) Experimental data (black) and the linlog simulation (blue). Units are the same as Figure 1. b) Comparison of the theoretical elasticities (black), with the linlog elasticities (white) estimated from steady state perturbation data.

In order to obtain better estimates of the elasticities, steady state perturbation data are introduced. The steady state enzyme perturbation data are generated as described in the Methods section (see section Steady state perturbations): in order to resolve the feed forward effect of FdP on the V_PK _reaction, the enzyme amount of the V_GAPD _reaction is perturbed in addition to the V_IN _and V_ATPase_. Using the steady state perturbation data in Table 1, and the calculation procedure described in the Methods section (see section Determining elasticities from steady state perturbation data), we have estimated the elasticities for the *in silico *network under study. After the estimation of the elasticities using steady state data only, we have also simulated the dynamic perturbation of Figure 1a using linlog kinetics, using the estimated elasticities. The results are presented in Figure 4. Figure 4a shows the experimental data and the linlog simulation of the same pulse, and Figure 4b compares the elasticities estimated using the steady state data with the theoretical elasticities. As can be seen from Figure 4b, using the steady state data, the elasticities are estimated in good agreement with the theoretical elasticities. However, as can be observed from Figure 4a, the linlog model deviates significantly from the experimental data, so the estimated elasticities have to be further refined.

**Table 1:** Relative effects of steady state perturbations on metabolite concentrations and rates. The metabolites and the rates are given relative to their reference states

**Perturbation Applied**			
	*e*_IN_/*e*_IN_^0 ^= 1.2	*e*_ATPase_/*e*_ATPase_^0 ^= 0.9	*e*_GAPD_/*e*_GAPD_^0 ^= 0.8

***Normalized metabolites***			

G^ext^'	0.793	1	1
G^in^'	1.128	0.999	1
G6P'	1.15	1.103	1
FdP'	1.093	0.959	1.369
PEP'	1.131	1.055	0.908
ATP'	1.045	1.071	1
Pol'	3.053	2.208	1
Gol'	1.077	0.983	1
EtOH'	1.077	0.983	1

***Normalized rates***			

V_IN_'	1.104	1	1
V_HK_'	1.104	1	1
V_PFK_'	1.077	0.983	1
V_GAPD_'	1.077	0.983	1
V_PK_'	1.077	0.983	1
V_Pol_'	3.053	2.208	1
V_Gol_'	1.077	0.983	1
V_ATPase_'	1.045	0.964	1

**Figure 5:**
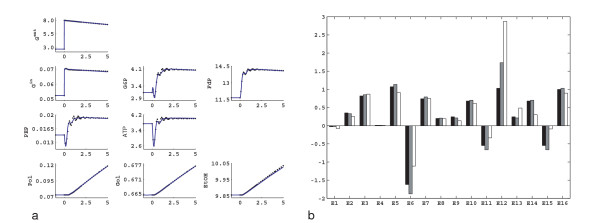
Results of the final estimation. a) Experimental data (black) and the linlog simulation using final elasticities (blue). Units are the same as Figure 1. b) Comparison of the theoretical elasticities (black), initial estimates from steady state perturbation data (grey) and the final estimates (white).

Having a good initial estimate for the linlog elasticities from the steady state perturbation data only, we used in the second step both the dynamic and steady state data for the parameter estimation procedure. The nonlinear regression procedure allows combining both available steady state and dynamic experimental data, so that all parameters can be accurately estimated. After the non-linear fit to the data, we have obtained the final estimation of the elasticities. The simulation of the pulse experiment with these final estimates, and the comparison of the elasticities are given in Figure 5a and Figure 5b respectively, which shows that most of the elasticities are correctly estimated and that the experimental data are well described.

### Analysis of parameter identifiability and model reduction

In the previous sections, we have seen that we could not determine all the parameters from dynamic perturbation data only and we have introduced the steady state perturbation data. After determination of all of the elasticities, using both types of perturbation data, we analyzed back the identifiability of each parameter under noise. In order to carry the identifiability and error propagation analysis, MC simulation was used. As described in the Methods section (see section Error propagation analysis), 10% relative error was added to the noise free data represented in Figure 1a (rapid sampling experiment), and the non-linear estimation procedure was implemented using the elasticities estimated from the steady state data, as the initial guesses. After repeating this scheme 50 times, we obtained a distribution for each of the elasticities from which we calculated the relative error for each elasticity as the standard deviation per mean of the corresponding elasticity (Table 2).

**Table 2:** The relative deviations of the elasticities, expressed as the standard deviation per mean*100, and the p-value at which the null hypothesis, that the actual elasticity is zero and that the estimated value was caused by the random error only, can not be rejected

Elasticity Parameter	Relative standard deviation σ2μ MathType@MTEF@5@5@+=feaafiart1ev1aaatCvAUfKttLearuWrP9MDH5MBPbIqV92AaeXatLxBI9gBaebbnrfifHhDYfgasaacH8akY=wiFfYdH8Gipec8Eeeu0xXdbba9frFj0=OqFfea0dXdd9vqai=hGuQ8kuc9pgc9s8qqaq=dirpe0xb9q8qiLsFr0=vr0=vr0dc8meaabaqaciaacaGaaeqabaqabeGadaaakeaadaWccaqaamaakaaabaacciGae83Wdm3aaWbaaSqabeaacqaIYaGmaaaabeaaaOqaaiab=X7aTbaaaaa@3172@·100 [%]	p-value
E1	235.3	2.1E-03
E2	9.1	0
E3	10.8	0
E4	295.4	1.0E-02
E5	15.7	0
E6	26.7	0
E7	12.1	0
E8	150.9	1.1E-05
E9	342.4	2.2E-02
E10	60.5	3.3E-16
E11	144.6	5.5E-06
E12	12.0	0
E13	143.0	4.5E-06
E14	159.9	2.7E-05
E15	360.2	2.8E-02
E16	84.5	2.3E-11

The adjustable parameter *λ *in equation (6) determines the relative importance of the different sources of data. In our case, these are dynamic and steady state data. Notice that during the MC simulations, noise is added only to dynamic data. For steady state perturbations, the number of data points that can be obtained is theoretically infinite. This implies that, considering the central limit theorem, the steady state data can, in principle, be considered as noise free. However, the aim of the MC simulations was to elucidate the potentially unidentifiable elasticities using dynamic perturbation data, and therefore we chose *λ *to be equal to zero. The other extreme (*λ *= 1) would suppress the effect of noise and wouldn't lead to detection of poorly identifiable elasticities. The values in between, are up to the choice of the modeler, depending on how many data points have been obtained from a dynamic pulse experiment, the standard deviations of the measurements, etc. We have implemented different values for the value of *λ*, but the outcomes of the MC simulation, i.e. which elasticities can hardly be identified, did not change qualitatively.

From the distribution of each elasticity, the p-value, indicating the probability that the actual elasticity is zero and that the estimated value was caused by the random error only, is calculated via t-test and the results are given in Table 2 (second column). The elasticities E1, E4, E9, and E15 have a relative standard deviation higher than 200%, so these elasticities are not identifiable under the 10% noise present. Therefore we conclude that these elasticities are clearly candidates for model reduction by assigning them to be zero (E1, E4, E9, E15 = 0).

A closer look reveals that E9 represents the feed-forward effect of FdP on V_PK_. E4 and E15 represent the effect of ATP on V_HK _and V_Gol _respectively and E1 is the feedback inhibition of G6P on V_IN_. Notice that the elasticities of the reactions of V_IN_, V_PK _and V_Gol _were already badly identified, when we estimated the elasticities using dynamic data only (Figure 2).

**Figure 6:**
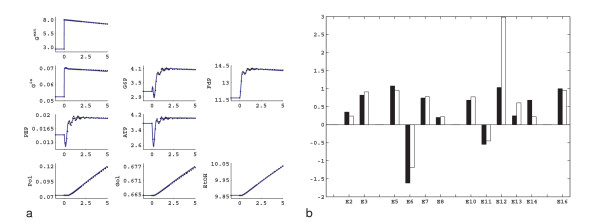
Results of the estimation of the elasticities, after model reduction. a) Experimental data (black) and the linlog simulation with the reduced elasticity matrix (blue). Units are the same as Figure 1. b) Comparison of the theoretical elasticities (black), with the linlog elasticities (white).

Using the arguments stated above, we set the values of these four (E1, E4, E9, E15) elasticities to zero, implemented the non-linear regression step, with the new sparser elasticity matrix, and estimated the elasticities using steady state and dynamic data. The results are presented in Figure 6a, where the experimental data and the linlog simulation using the new set of elasticities are given and in Figure 6b where the comparison of the estimated elasticities with the theoretical ones is presented. It is clear that with the new set of 12 elasticities (replacing the original 16) the dynamic pulse can also be simulated in good agreement with the experimental data. The value of the objective function increased only by 0.04%, when the degrees of freedom increased by 21% (degrees of freedom increased from 19 (= 35 data points – 16 parameters), to 23 (= 35 data points – 12 parameters)) showing that elimination of these four elasticities in fact improved the quality of the fit.

### Cross validation of the obtained model

**Figure 7:**
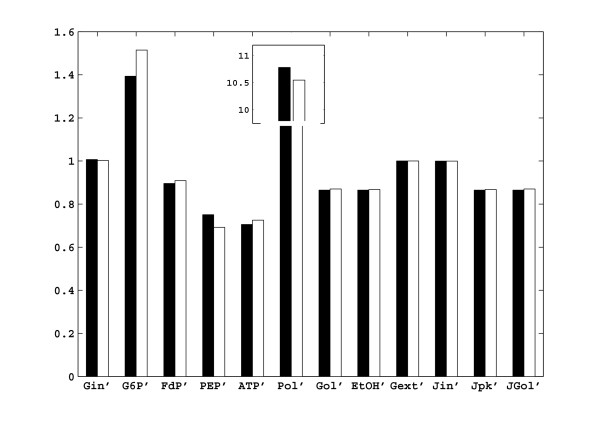
Results of the cross validation study. An independent steady state perturbation is introduced, in which the activity of V_PFK _is decreased by half (see text). A comparison of the normalized *in silico *experimental results (black) with normalized model prediction (white) are given. The concentrations are normalized with respect to the reference conditions, given in Table 3.

In order to validate the estimated elasticities, we have performed a cross validation study, for which we have generated an independent dataset, consisting of steady state perturbation data, and we have predicted the effect of that perturbation with our estimated parameters, and compared the results. Since we already perturbed V_IN_, V_GAPD _and V_ATPase _and included the resulting data in the identification dataset (Table 1), we had to select another enzyme in the pathway as the target for independent steady state perturbation. Among the remaining enzymes, we chose to inhibit the activity of V_PFK _by 50%, because this enzyme assumes many interactions and is known to be a complex enzyme, which would be a challenge to cross validate for our network. The comparison between the model prediction and the experimental results is given in Figure 7. The level of extracellular and intracellular glucose did not change, whereas the levels of G6P and polysaccharides increased considerably and the levels of PEP, FdP, ethanol and glycerol decreased. The linlog model predicted this steady state perturbation with maximal deviation of only 8.5% in G6P. We conclude that the linlog model with the estimated parameters performed satisfactorily for the test case considered.

### Calculation of the systemic properties

**Figure 8:**
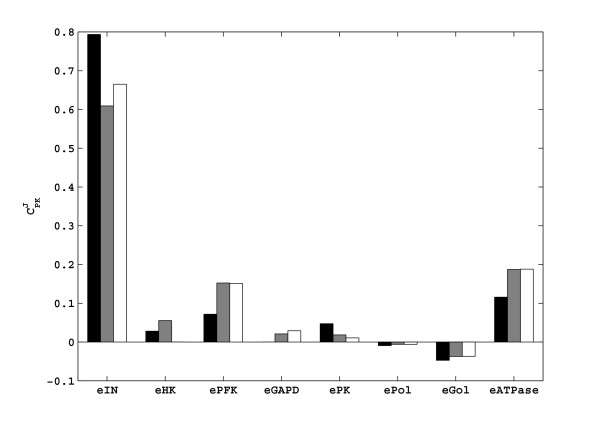
Comparison of the systemic properties, flux control coefficients (**C**^**J0**^) of the ethanol flux. First column (black): theoretical **C**^**J0**^s calculated using theoretical elasticities. Second column (grey): **C**^**J0**^s calculated using estimated elasticities in Figure 5b. Third column (white): **C**^**J0**^s calculated using the estimated elasticities of the reduced model (Figure 6b).

After the estimation of the elasticities, we have calculated the flux control coefficients (**C**^**J0**^) using equation (11). The comparison of these with the theoretical control coefficients of the main flux (*J*_PK_) is presented in Figure 8. The flux is controlled mainly by three enzymes: the hexose transporter (*e*_*IN*_), and to a lesser extent by the phosphofructokinase (*e*_*PFK*_), and the ATPase (*e*_*ATPase*_). These findings are in qualitative agreement with the literature [31]. It is noticeable that the elimination of the four elasticities did not change these results significantly; the same three reactions still have the main control of the glycolytic flux.

**Figure 9:**
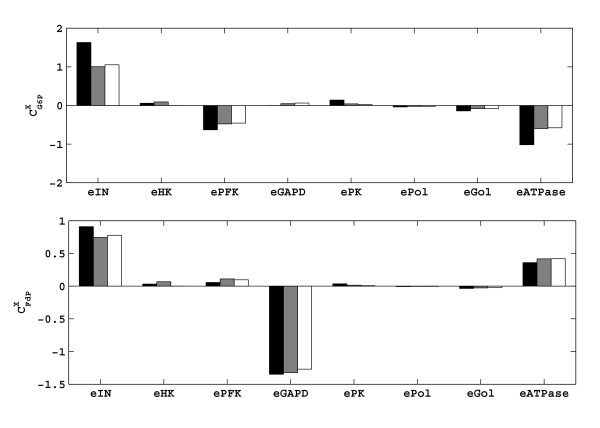
Comparison of the systemic properties, concentration control coefficients (**C**^**x0**^) of the two branch point metabolites: G6P (upper panel) and FdP (lower panel). First column (black): theoretical **C**^**x0**^s calculated using theoretical elasticities. Second column (grey): **C**^**x0**^s calculated using estimated elasticities in Figure 5b. Third column (white): **C**^**x0**^s calculated using the estimated elasticities of the reduced model (Figure 6b).

The concentration control coefficients (**C**^**x0**^) are also calculated using equation (10). Figure 9 gives the comparison of the **C**^**x0**^s of the two branch point metabolites, G6P and FdP. The levels of both metabolites can be increased by increasing the activity of the hexose transporter. Increasing the activity of *e*_ATPase _will decrease the level of G6P, whereas it increases the level of FdP. Increasing the activity of *e*_PFK _will decrease and increase the levels of G6P and FdP respectively. Lastly, an increase in the activity of *e*_GAPD _will result in a slight increase in G6P level and a decrease in FdP level. These results are also in agreement with the previous studies. As expected, the model reduction did not have a significant effect on the estimated control coefficients.

## Discussion

Large mathematical models are needed for finding the targets for engineering realistic metabolic systems. Currently, the available large models of several organisms e.g. *Saccharomyces cerevisiae *are mostly stoichiometric models. Although these models are highly relevant for knockout studies and determining the capabilities of the network considered, they have some limitations, e.g., they cannot predict the time courses, do not give insights in molecular events and are based on the "assumed goals" of the cells. Hence, for driving engineering interventions, kinetic models are needed. Here, we have presented a method to estimate the elasticities using the linlog kinetic format, from a combination of steady state and dynamic pulse experiments. In the past, there have been efforts to extract MCA parameters from transient metabolite data. Liao and Delgado presented a method to obtain the control coefficients from such data [34]. Later, it was shown by Elde and Zacchi through MC simulations that this method is highly sensitive to noise [35]. Moreover a full dynamic kinetic model is not obtained. Here, we have described an estimation procedure which directly yields the elasticities as parameters instead of control coefficients, hence allowing the construction of a full kinetic model. Subsequently, the control coefficients can be obtained from the summation and connectivity theorems. Such a full kinetic model can also be used to simulate the effect of different perturbations on metabolism of a microorganism in a fermentor.

The method presented here assumes the availability of dynamic perturbation data. Although the list of metabolites is growing, not all of the metabolites in the cell can be measured. In a recent study, Wang and coworkers described an extension of the MCA, under uncertainty [36-38] in which the authors described a framework to calculate the control coefficients when either there are no measurements on metabolites, or the available measurements are subject to high uncertainty. Their proposed framework can be considered as complementary to the method presented in this paper. In a case where accurate measurements are available, the present paper provides a mathematical approach to obtain the elasticities as kinetic parameters, from which one can calculate the control coefficients.

When compared to previous studies where linlog kinetics have been applied in combination with highly idealized linear pathways or small networks with branch points, the model in this work represents a more realistic case. The model used in this study represents an intermediate size system with branch points and conserved moieties. The proposed method can easily be extended to larger networks that are needed not only to understand the functioning of living cells, but also to infer engineering applications of e.g. microorganisms.

In the example considered in this paper, we have assumed that the zero entries of the elasticity matrix are known. This is a reasonable assumption, since numerous enzymes in the primary metabolism of many organisms are extensively studied and there is a dedicated public compendium for such information [39], allowing the use of *a priori *knowledge on elasticities which are zero.

In the absence of any *a priori *knowledge on the zero elasticities, the elasticities have to be estimated from the full elasticity matrix, for which vastly more experimental data are needed. In the MCA literature, there are several attempts to determine the different perturbations needed in order to determine all elasticities [22,23]. However, one needs to consider that an enzyme will never be affected by all metabolites; there is a physical limit for e.g. the maximum number of binding sites of each enzyme in a biological network.

From the results presented here, it has been shown that, despite the rich information content of the data obtained from dynamic experiments, not every elasticity of the network could be correctly estimated. This problem of parameter identifiability would be much more pronounced in a case where all (possible) interactions are taken into account, caused by the combinatorial explosion of number of parameters to be estimated. In the current work, in order to resolve some of the interactions which could not be resolved from the dynamic data, steady state enzyme perturbations have been introduced. In order to get full kinetic models of microorganisms, accurate measurements of metabolite concentrations resulting from independent perturbations are needed, such as presented in [28] and [40]. At this point it is important to state that the property of the linlog kinetic format, that the rate equations are linear in the kinetic parameters, allows simultaneous use of alternative datasets by concatenating them in one parameter estimation scheme, i.e. it is straightforward to extend the data matrix **Y **in equation (9) and the vector *χ*_*s*im _in equation (6), with the data from alternative dynamic and steady state perturbation experiments. In addition, the decoupling of the parameters is also immediate i.e. we can isolate poorly identifiable elasticities and estimate the remaining ones accurately and thereby reduce the problem.

It is worth mentioning that the obtained parameters (elasticities) are true kinetic parameters that reflect the properties of the enzymes with respect to the corresponding metabolites. They can be assumed to remain invariant as long as the enzyme keeps its properties in response to the changes in the environmental conditions. Additionally, since the linlog kinetics provides an approximation to the actual rate of the corresponding reaction over certain interval, the elasticity parameter that performs best may be different than the theoretical value of the corresponding elasticity. This should be kept in mind when comparing some of the less well determined elasticity values to the theoretical ones.

## Conclusion

Constructing dynamic models of metabolic reaction networks under *in vivo *conditions using data obtained from perturbation experiments remains still a challenging problem in the area of systems biology. In this contribution, we presented a method which allows the determination of the elasticities directly from experimental data from typical dynamic and/or steady state perturbation experiments. These elasticities allow the reconstruction of the full kinetic model of the glycolysis of *Saccharomyces cerevisiae*, and the determination of the control coefficients. We further show by *a posteriori *parameter identifiability analysis that a subset of elasticities could not be identified using dynamic perturbation data only. Introduction of additional experimental information, i.e. steady state experiments, solved this parameter identification problem.

Considering the description of the dynamics, results of the cross validation studies and the final values of the elasticities, we conclude that linlog kinetics, although being an approximate kinetic format, performs very satisfactorily for estimating elasticities from data obtained from dynamic simulation of a mechanistic model, that was realistic in terms of biochemical complexity (glycolysis), noise added and sampling frequency.

## Methods

In linlog kinetics, all rate equations have the same mathematical structure: proportionality to the enzyme level and linearity in the parameters (elasticities, εxv
 MathType@MTEF@5@5@+=feaafiart1ev1aaatCvAUfKttLearuWrP9MDH5MBPbIqV92AaeXatLxBI9gBaebbnrfifHhDYfgasaacH8akY=wiFfYdH8Gipec8Eeeu0xXdbba9frFj0=OqFfea0dXdd9vqai=hGuQ8kuc9pgc9s8qqaq=dirpe0xb9q8qiLsFr0=vr0=vr0dc8meaabaqaciaacaGaaeqabaqabeGadaaakeaaiiGacqWF1oqzdaqhaaWcbaGaemiEaGhabaGaemODayhaaaaa@3175@ and εcv
 MathType@MTEF@5@5@+=feaafiart1ev1aaatCvAUfKttLearuWrP9MDH5MBPbIqV92AaeXatLxBI9gBaebbnrfifHhDYfgasaacH8akY=wiFfYdH8Gipec8Eeeu0xXdbba9frFj0=OqFfea0dXdd9vqai=hGuQ8kuc9pgc9s8qqaq=dirpe0xb9q8qiLsFr0=vr0=vr0dc8meaabaqaciaacaGaaeqabaqabeGadaaakeaaiiGacqWF1oqzdaqhaaWcbaGaem4yamgabaGaemODayhaaaaa@314B@) as represented by equation (2). This equation can be generalized in vector form to represent the rate vector of the metabolic network under consideration

**v **= **J**^**0**^·**e'**·(**i **+ **E**^**x **^ln(**x'**) + **E**^**c **^ln(**c'**))     (3)

where the vector **v **is the (*r *× 1) rate vector, r being the number of rates, **J**^**0 **^is the square diagonal matrix containing the reference state fluxes (**J**^**0 **^= diag(Ji0
 MathType@MTEF@5@5@+=feaafiart1ev1aaatCvAUfKttLearuWrP9MDH5MBPbIqV92AaeXatLxBI9gBaebbnrfifHhDYfgasaacH8akY=wiFfYdH8Gipec8Eeeu0xXdbba9frFj0=OqFfea0dXdd9vqai=hGuQ8kuc9pgc9s8qqaq=dirpe0xb9q8qiLsFr0=vr0=vr0dc8meaabaqaciaacaGaaeqabaqabeGadaaakeaacqWGkbGsdaqhaaWcbaGaemyAaKgabaGaeGimaadaaaaa@303F@) *i *= 1,...,*r*), **e' **is the square diagonal matrix containing relative enzyme levels (**e' **= diag(*e*_*i*_/ei0
 MathType@MTEF@5@5@+=feaafiart1ev1aaatCvAUfKttLearuWrP9MDH5MBPbIqV92AaeXatLxBI9gBaebbnrfifHhDYfgasaacH8akY=wiFfYdH8Gipec8Eeeu0xXdbba9frFj0=OqFfea0dXdd9vqai=hGuQ8kuc9pgc9s8qqaq=dirpe0xb9q8qiLsFr0=vr0=vr0dc8meaabaqaciaacaGaaeqabaqabeGadaaakeaacqWGLbqzdaqhaaWcbaGaemyAaKgabaGaeGimaadaaaaa@3075@)*i *= 1,...,*r*), **i **is the (*r *× 1) vector of ones, **E**^**x **^and **E**^**c **^are the (*r *× *m*_*x*_) and (*r *× *m*_*c*_) elasticity matrices, *m*_*x *_and *m*_*c *_being the number of intracellular and extracellular metabolites respectively, and **x' **and **c' **are the (*m*_*x *_× 1) and (*m*_*c *_× 1) vectors containing relative concentrations of the intracellular and extracellular metabolites respectively (**x' **= *x*_*j*_/xj0
 MathType@MTEF@5@5@+=feaafiart1ev1aaatCvAUfKttLearuWrP9MDH5MBPbIqV92AaeXatLxBI9gBaebbnrfifHhDYfgasaacH8akY=wiFfYdH8Gipec8Eeeu0xXdbba9frFj0=OqFfea0dXdd9vqai=hGuQ8kuc9pgc9s8qqaq=dirpe0xb9q8qiLsFr0=vr0=vr0dc8meaabaqaciaacaGaaeqabaqabeGadaaakeaacqWG4baEdaqhaaWcbaGaemOAaOgabaGaeGimaadaaaaa@309D@*j *= 1,...,*m*_*x *_and **c' **= *x*_*k*_/xk0
 MathType@MTEF@5@5@+=feaafiart1ev1aaatCvAUfKttLearuWrP9MDH5MBPbIqV92AaeXatLxBI9gBaebbnrfifHhDYfgasaacH8akY=wiFfYdH8Gipec8Eeeu0xXdbba9frFj0=OqFfea0dXdd9vqai=hGuQ8kuc9pgc9s8qqaq=dirpe0xb9q8qiLsFr0=vr0=vr0dc8meaabaqaciaacaGaaeqabaqabeGadaaakeaacqWG4baEdaqhaaWcbaGaem4AaSgabaGaeGimaadaaaaa@309F@*k *= 1,...,*m*_*c*_). In each term, the superscript '^0^' indicates the reference state values.

### Determining elasticities from steady state perturbation data

Given that for steady state perturbation experiments, we obtain information on fluxes (*J*_*i*_'s) using the mass balances and the measured metabolite concentrations (*x*_*j*_), we can directly use the equation (3) for the estimation of the elasticities. When it is rearranged, the equation (3) can be presented in the following standard linear model:

**a **= **Y·b **    (4)

where **a **is the (*r *× 1) measurement vector that contains the measured normalized fluxes and normalized enzyme levels (**a **= (**J**^**0**^**e'**)^-1 ^**v **- **i**), **b **the (*p *× 1) vector that contains the non-zero elasticities of the original elasticity matrices **E**^**x **^and **E**^**c**^, and **Y **is the (*r *× *p*) design matrix of which each i^th ^row contains as nonzero elements ln(*x*_*j*_/xj0
 MathType@MTEF@5@5@+=feaafiart1ev1aaatCvAUfKttLearuWrP9MDH5MBPbIqV92AaeXatLxBI9gBaebbnrfifHhDYfgasaacH8akY=wiFfYdH8Gipec8Eeeu0xXdbba9frFj0=OqFfea0dXdd9vqai=hGuQ8kuc9pgc9s8qqaq=dirpe0xb9q8qiLsFr0=vr0=vr0dc8meaabaqaciaacaGaaeqabaqabeGadaaakeaacqWG4baEdaqhaaWcbaGaemOAaOgabaGaeGimaadaaaaa@309D@) and ln(*c*_*k*_/ck0
 MathType@MTEF@5@5@+=feaafiart1ev1aaatCvAUfKttLearuWrP9MDH5MBPbIqV92AaeXatLxBI9gBaebbnrfifHhDYfgasaacH8akY=wiFfYdH8Gipec8Eeeu0xXdbba9frFj0=OqFfea0dXdd9vqai=hGuQ8kuc9pgc9s8qqaq=dirpe0xb9q8qiLsFr0=vr0=vr0dc8meaabaqaciaacaGaaeqabaqabeGadaaakeaacqWGJbWydaqhaaWcbaGaem4AaSgabaGaeGimaadaaaaa@3075@) at positions corresponding with the non-zero elasticities εxjvi
 MathType@MTEF@5@5@+=feaafiart1ev1aaatCvAUfKttLearuWrP9MDH5MBPbIqV92AaeXatLxBI9gBaebbnrfifHhDYfgasaacH8akY=wiFfYdH8Gipec8Eeeu0xXdbba9frFj0=OqFfea0dXdd9vqai=hGuQ8kuc9pgc9s8qqaq=dirpe0xb9q8qiLsFr0=vr0=vr0dc8meaabaqaciaacaGaaeqabaqabeGadaaakeaaiiGacqWF1oqzdaqhaaWcbaGaemiEaG3aaSbaaWqaaiabdQgaQbqabaaaleaacqWG2bGDdaWgaaadbaGaemyAaKgabeaaaaaaaa@3492@ and εckvi
 MathType@MTEF@5@5@+=feaafiart1ev1aaatCvAUfKttLearuWrP9MDH5MBPbIqV92AaeXatLxBI9gBaebbnrfifHhDYfgasaacH8akY=wiFfYdH8Gipec8Eeeu0xXdbba9frFj0=OqFfea0dXdd9vqai=hGuQ8kuc9pgc9s8qqaq=dirpe0xb9q8qiLsFr0=vr0=vr0dc8meaabaqaciaacaGaaeqabaqabeGadaaakeaaiiGacqWF1oqzdaqhaaWcbaGaem4yam2aaSbaaWqaaiabdUgaRbqabaaaleaacqWG2bGDdaWgaaadbaGaemyAaKgabeaaaaaaaa@346A@ in vector **b**. The equation (4) can be solved to obtain the elasticities using linear regression according to:

**b **= (**Y**^**T**^**Y**)^-1 ^**Y**^**T**^·**a **    (5)

This shows that estimation of elasticities from steady state perturbations requires data on metabolite levels (ln(*x*_*j*_/xj0
 MathType@MTEF@5@5@+=feaafiart1ev1aaatCvAUfKttLearuWrP9MDH5MBPbIqV92AaeXatLxBI9gBaebbnrfifHhDYfgasaacH8akY=wiFfYdH8Gipec8Eeeu0xXdbba9frFj0=OqFfea0dXdd9vqai=hGuQ8kuc9pgc9s8qqaq=dirpe0xb9q8qiLsFr0=vr0=vr0dc8meaabaqaciaacaGaaeqabaqabeGadaaakeaacqWG4baEdaqhaaWcbaGaemOAaOgabaGaeGimaadaaaaa@309D@) and ln(*c*_*k*_/ck0
 MathType@MTEF@5@5@+=feaafiart1ev1aaatCvAUfKttLearuWrP9MDH5MBPbIqV92AaeXatLxBI9gBaebbnrfifHhDYfgasaacH8akY=wiFfYdH8Gipec8Eeeu0xXdbba9frFj0=OqFfea0dXdd9vqai=hGuQ8kuc9pgc9s8qqaq=dirpe0xb9q8qiLsFr0=vr0=vr0dc8meaabaqaciaacaGaaeqabaqabeGadaaakeaacqWGJbWydaqhaaWcbaGaem4AaSgabaGaeGimaadaaaaa@3075@) presented in matrix **Y**) enzyme activities (presented in matrix **e'**) and steady state fluxes (presented in the vector **v **and the matrix **J**^**0**^).

### Determining elasticities from dynamic perturbation data

Since in a dynamic perturbation experiment, the rate information can not be obtained directly, we will follow different procedure here and we will only make use of the time profiles of the measured metabolites (*x*_*i*_'s) in treating dynamic perturbation data. The elasticities are, in this case, estimated via non-linear parameter estimation procedure, in which the objective function to be minimized is the weighed squared error between the experimental response and simulation results. The general form of the objective function is given in equation(6):

objfun=(1−λ)∑i=1mγi⋅(∑j=1q(χexp⁡,i,j−χsim,i,jx0,i)2)+λ⋅∑k=1r(J'k,exp⁡−J'k,simJk0)20≤λ≤1     (6)
 MathType@MTEF@5@5@+=feaafiart1ev1aaatCvAUfKttLearuWrP9MDH5MBPbIqV92AaeXatLxBI9gBaebbnrfifHhDYfgasaacH8akY=wiFfYdH8Gipec8Eeeu0xXdbba9frFj0=OqFfea0dXdd9vqai=hGuQ8kuc9pgc9s8qqaq=dirpe0xb9q8qiLsFr0=vr0=vr0dc8meaabaqaciaacaGaaeqabaqabeGadaaakeaafaqabeqacaaabaGaee4Ba8MaeeOyaiMaeeOAaOMaeeOzayMaeeyDauNaeeOBa4Maeyypa0ZaaeWaaeaacqaIXaqmcqGHsisliiGacqWF7oaBaiaawIcacaGLPaaadaaeWbqaaiab=n7aNnaaBaaaleaacqWGPbqAaeqaaOGaeyyXIC9aaeWaaeaadaaeWbqaamaabmaabaWaaSaaaeaacqWFhpWydaWgaaWcbaGagiyzauMaeiiEaGNaeiiCaaNaeiilaWIaemyAaKMaeiilaWIaemOAaOgabeaakiabgkHiTiab=D8aJnaaBaaaleaacqWGZbWCcqWGPbqAcqWGTbqBcqGGSaalcqWGPbqAcqGGSaalcqWGQbGAaeqaaaGcbaGaemiEaG3aaSbaaSqaaiabicdaWiabcYcaSiabdMgaPbqabaaaaaGccaGLOaGaayzkaaWaaWbaaSqabeaacqaIYaGmaaaabaGaemOAaOMaeyypa0JaeGymaedabaGaemyCaehaniabggHiLdaakiaawIcacaGLPaaaaSqaaiabdMgaPjabg2da9iabigdaXaqaaiabd2gaTbqdcqGHris5aOGaey4kaSIae83UdWMaeyyXIC9aaabCaeaadaqadaqaamaalaaabaGaemOsaOKaei4jaCYaaSbaaSqaaiabdUgaRjabcYcaSiGbcwgaLjabcIha4jabcchaWbqabaGccqGHsislcqWGkbGscqGGNaWjdaWgaaWcbaGaem4AaSMaeiilaWIaem4CamNaemyAaKMaemyBa0gabeaaaOqaaiabdQeaknaaDaaaleaacqWGRbWAaeaacqaIWaamaaaaaaGccaGLOaGaayzkaaWaaWbaaSqabeaacqaIYaGmaaaabaGaem4AaSMaeyypa0JaeGymaedabaGaemOCaihaniabggHiLdaakeaacqaIWaamcqGHKjYOcqWF7oaBcqGHKjYOcqaIXaqmaaGaaCzcaiaaxMaadaqadaqaaiabiAda2aGaayjkaiaawMcaaaaa@9CC8@

the limits of the summations, *m*, *q *and *r *are the number of metabolites, measured time points and fluxes respectively. In equation (6), *χ*_exp _is the experimentally measured, *χ*_sim _is the simulated (using linlog kinetics) metabolite matrix containing intracellular and extracellular metabolite concentrations. Two additional parameters are introduced here:

*γ *to weigh the effect of uncertainty in different metabolites and *λ *to weigh the effect of two different sources of data, namely steady state and dynamic perturbation data. In the second term of the equation(6), *J*'_*sim *_and *J*'_exp _represent the experimental and simulated steady state fluxes. The equation (6) contains the rate information implicitly, since the *χ*_sim _results from the integration of the set of ode's representing the dynamics of the system. Note also that although we already explained the treatment of the steady state perturbation data in the previous section, we explicitly included the second term in equation (6) again; to state clearly that this form of the objective function allows also the integration of data from different types of experiments.

Two main classes of optimization algorithms are available to minimize the objective function in equation (6): greedy algorithms and evolutionary algorithms. Moles *et al*. presented a comparison of global optimization methods used for parameter estimation in biochemical pathways [41]. In that review, they discussed various global optimization methods and concluded that the algorithm that uses evolutionary strategy using stochastic ranking performed best. On the other hand, they also pointed out that, generally, evolutionary algorithms require high computational effort. The alternative, greedy algorithms, are fast, but in turn require an initial estimate close to the optimal solution. A good initial estimate is necessary not only to evade local minima and improve the solution performance, (i.e. convergence time, finding a global optimum) but also to prevent highly stiff systems, which increase the computation time. In this work, we chose to use a greedy (simplex) algorithm, mainly because the linlog kinetic format has the advantage to provide a good initial estimate that can be obtained directly from the experimental data via linear regression (see below) so that the method presented in this paper does not require the robustness of the evolutionary algorithms towards the initial estimate. It is noteworthy that alternative approximative kinetic formats such as the two proposed formats of BST (i.e. GMA or S-system forms) lack this advantage of providing a good initial guess to the non-linear regression step. With these formats, zero is generally assumed as the initial guess for the non-linear parameter estimation problem [42].

To obtain initial estimates, we start with the general dynamic model of a metabolic system in a typical rapid pulse experiment in a chemostat which is given by the mass balances for the *m*_*x *_intracellular (**x**) and *m*_*c *_extracellular (**c**) metabolites:

dxdt=S⋅v−μ⋅xdcdt=D⋅(cfeed−c)+Sc⋅v⋅cX     (7)
 MathType@MTEF@5@5@+=feaafiart1ev1aaatCvAUfKttLearuWrP9MDH5MBPbIqV92AaeXatLxBI9gBaebbnrfifHhDYfgasaacH8akY=wiFfYdH8Gipec8Eeeu0xXdbba9frFj0=OqFfea0dXdd9vqai=hGuQ8kuc9pgc9s8qqaq=dirpe0xb9q8qiLsFr0=vr0=vr0dc8meaabaqaciaacaGaaeqabaqabeGadaaakeaafaqaaeGabaaabaWaaSaaaeaacqWGKbazcqWH4baEaeaacqWGKbazcqWG0baDaaGaeyypa0JaeC4uamLaeyyXICTaeCODayNaeyOeI0ccciGae8hVd0MaeyyXICTaeCiEaGhabaWaaSaaaeaacqWGKbazcqWHJbWyaeaacqWGKbazcqWG0baDaaGaeyypa0JaemiraqKaeyyXIC9aaeWaaeaacqWHJbWydaWgaaWcbaGaemOzayMaemyzauMaemyzauMaemizaqgabeaakiabgkHiTiabhogaJbGaayjkaiaawMcaaiabgUcaRiabhofatnaaBaaaleaacqWGJbWyaeqaaOGaeyyXICTaeCODayNaeyyXICTaem4yam2aaSbaaSqaaiabbIfaybqabaaaaOGaaCzcaiaaxMaadaqadaqaaiabiEda3aGaayjkaiaawMcaaaaa@6366@

Where **x **and **c **are the (*m*_*x *_× 1) and (*m*_*c *_× 1) vectors containing concentrations of intracellular and extracellular metabolites respectively, expressed in *μ*mol gDW^-1 ^and *μ*mol L^-1 ^respectively, **S **and **S**_**c **_are (*m*_*x *_× *r*) and (*m*_*c *_× *r*) stoichiometric matrices for the intra and extracellular metabolites respectively; **v **is the (*r *× 1) biomass specific reaction rate vector in *μ*mol gDW^-1 ^hr^-1^, **c**_*feed *_is the (*m*_*c *_× 1) vector containing concentration of the extracellular metabolites in the feed expressed in *μ*mol L^-1^, *D *is the dilution rate (hr^-1^), *μ *is the biomass specific growth rate (hr^-1^) and *c*_*X *_is the biomass concentration in gDW L^-1^.

Substitution of **v **by linlog kinetic rate equation (3) in the mass balance equation (7) and assuming that there is no change in the enzyme levels (**e' **= **I**_*r *× *r*_) yields:

dxdt=S⋅J0⋅(i+Ex⋅ln⁡(x')+Ec⋅ln⁡(c'))−μ⋅xdcdt=D⋅(cfeed−c)+Sc⋅J0⋅(i+Ex⋅ln⁡(x')+Ec⋅ln⁡(c'))⋅cX     (8)
 MathType@MTEF@5@5@+=feaafiart1ev1aaatCvAUfKttLearuWrP9MDH5MBPbIqV92AaeXatLxBI9gBaebbnrfifHhDYfgasaacH8akY=wiFfYdH8Gipec8Eeeu0xXdbba9frFj0=OqFfea0dXdd9vqai=hGuQ8kuc9pgc9s8qqaq=dirpe0xb9q8qiLsFr0=vr0=vr0dc8meaabaqaciaacaGaaeqabaqabeGadaaakeaafaqaaeGabaaabaWaaSaaaeaacqWGKbazcqWH4baEaeaacqWGKbazcqWG0baDaaGaeyypa0JaeC4uamLaeyyXICTaeCOsaO0aaWbaaSqabeaacqWHWaamaaGccqGHflY1daqadaqaaiabhMgaPjabgUcaRiabhweafnaaCaaaleqabaGaeCiEaGhaaOGaeyyXICTagiiBaWMaeiOBa42aaeWaaeaacqWH4baEcqWHNaWjaiaawIcacaGLPaaacqGHRaWkcqWHfbqrdaahaaWcbeqaaiabhogaJbaakiabgwSixlGbcYgaSjabc6gaUnaabmaabaGaeC4yamMaei4jaCcacaGLOaGaayzkaaaacaGLOaGaayzkaaGaeyOeI0ccciGae8hVd0MaeyyXICTaeCiEaGhabaWaaSaaaeaacqWGKbazcqWHJbWyaeaacqWGKbazcqWG0baDaaGaeyypa0JaemiraqKaeyyXIC9aaeWaaeaacqWHJbWydaWgaaWcbaGaemOzayMaemyzauMaemyzauMaemizaqgabeaakiabgkHiTiabhogaJbGaayjkaiaawMcaaiabgUcaRiabhofatnaaBaaaleaacqWGJbWyaeqaaOGaeyyXICTaeCOsaO0aaWbaaSqabeaacqWHWaamaaGccqGHflY1daqadaqaaiabhMgaPjabgUcaRiabhweafnaaCaaaleqabaGaeCiEaGhaaOGaeyyXICTagiiBaWMaeiOBa42aaeWaaeaacqWH4baEcqGGNaWjaiaawIcacaGLPaaacqGHRaWkcqWHfbqrdaahaaWcbeqaaiabhogaJbaakiabgwSixlGbcYgaSjabc6gaUnaabmaabaGaeC4yamMaei4jaCcacaGLOaGaayzkaaaacaGLOaGaayzkaaGaeyyXICTaem4yam2aaSbaaSqaaiabdIfaybqabaaaaOGaaCzcaiaaxMaadaqadaqaaiabiIda4aGaayjkaiaawMcaaaaa@A127@

After rearrangement (and taking into account that due to steady state of the reference, **S**·**J**^**0**^·**i **= **0**), the set of equations are integrated for each metabolite, from t_i _to t_i+1_,

Δx+μ⋅∫titi+1xdt=S⋅J0⋅[Ex Ec]⋅∫titi+1[ln⁡(x')ln⁡(c')]dt
 MathType@MTEF@5@5@+=feaafiart1ev1aaatCvAUfKttLearuWrP9MDH5MBPbIqV92AaeXatLxBI9gBaebbnrfifHhDYfgasaacH8akY=wiFfYdH8Gipec8Eeeu0xXdbba9frFj0=OqFfea0dXdd9vqai=hGuQ8kuc9pgc9s8qqaq=dirpe0xb9q8qiLsFr0=vr0=vr0dc8meaabaqaciaacaGaaeqabaqabeGadaaakeaacqqHuoarcqWH4baEcqGHRaWkiiGacqWF8oqBcqGHflY1daWdXaqaaiabhIha4jabdsgaKjabdsha0bWcbaGaemiDaq3aaSbaaWqaaiabdMgaPbqabaaaleaacqWG0baDdaWgaaadbaGaemyAaKMaey4kaSIaeGymaedabeaaa0Gaey4kIipakiabg2da9iabhofatjabgwSixlabhQeaknaaCaaaleqabaGaeCimaadaaOGaeyyXIC9aamWaaeaacqWHfbqrdaahaaWcbeqaaiabhIha4baakiaaykW7cqWHfbqrdaahaaWcbeqaaiabhogaJbaaaOGaay5waiaaw2faaiabgwSixpaapedabaWaamWaaqaabeqaaiGbcYgaSjabc6gaUnaabmaabaGaeCiEaGNaei4jaCcacaGLOaGaayzkaaaabaGagiiBaWMaeiOBa42aaeWaaeaacqWHJbWycqGGNaWjaiaawIcacaGLPaaaaaGaay5waiaaw2faaiabdsgaKjabdsha0bWcbaGaemiDaq3aaSbaaWqaaiabdMgaPbqabaaaleaacqWG0baDdaWgaaadbaGaemyAaKMaey4kaSIaeGymaedabeaaa0Gaey4kIipaaaa@72CB@

Δc−(D⋅∫titi+1(cfeed−c)dt+Sc⋅J0⋅i⋅cX⋅Δt)=Sc⋅J0⋅[Ex Ec]⋅∫titi+1[ln⁡(x')ln⁡(c')]dt⋅cX
 MathType@MTEF@5@5@+=feaafiart1ev1aaatCvAUfKttLearuWrP9MDH5MBPbIqV92AaeXatLxBI9gBaebbnrfifHhDYfgasaacH8akY=wiFfYdH8Gipec8Eeeu0xXdbba9frFj0=OqFfea0dXdd9vqai=hGuQ8kuc9pgc9s8qqaq=dirpe0xb9q8qiLsFr0=vr0=vr0dc8meaabaqaciaacaGaaeqabaqabeGadaaakeaacqqHuoarcqWHJbWycqGHsisldaqadaqaaiabdseaejabgwSixpaapedabaWaaeWaaeaacqWHJbWydaWgaaWcbaGaemOzayMaemyzauMaemyzauMaemizaqgabeaakiabgkHiTiabhogaJbGaayjkaiaawMcaaiabdsgaKjabdsha0bWcbaGaemiDaq3aaSbaaWqaaiabdMgaPbqabaaaleaacqWG0baDdaWgaaadbaGaemyAaKMaey4kaSIaeGymaedabeaaa0Gaey4kIipakiabgUcaRiabhofatnaaBaaaleaacqWGJbWyaeqaaOGaeyyXICTaeCOsaO0aaWbaaSqabeaacqWHWaamaaGccqGHflY1cqWHPbqAcqGHflY1cqWGJbWydaWgaaWcbaGaemiwaGfabeaakiabgwSixlabfs5aejabdsha0bGaayjkaiaawMcaaiabg2da9iabhofatnaaBaaaleaacqWGJbWyaeqaaOGaeyyXICTaeCOsaO0aaWbaaSqabeaacqWHWaamaaGccqGHflY1daWadaqaaiabhweafnaaCaaaleqabaGaeCiEaGhaaOGaaGPaVlabhweafnaaCaaaleqabaGaeC4yamgaaaGccaGLBbGaayzxaaGaeyyXIC9aa8qmaeaadaWadaabaeqabaGagiiBaWMaeiOBa42aaeWaaeaacqWH4baEcqGGNaWjaiaawIcacaGLPaaaaeaacyGGSbaBcqGGUbGBdaqadaqaaiabhogaJjabcEcaNaGaayjkaiaawMcaaaaacaGLBbGaayzxaaGaemizaqMaemiDaqhaleaacqWG0baDdaWgaaadbaGaemyAaKgabeaaaSqaaiabdsha0naaBaaameaacqWGPbqAcqGHRaWkcqaIXaqmaeqaaaqdcqGHRiI8aOGaeyyXICTaem4yam2aaSbaaSqaaiabdIfaybqabaaaaa@9932@

This can be presented in the following standard linear model:

[axac]=[YxYc]⋅b     (9)
 MathType@MTEF@5@5@+=feaafiart1ev1aaatCvAUfKttLearuWrP9MDH5MBPbIqV92AaeXatLxBI9gBaebbnrfifHhDYfgasaacH8akY=wiFfYdH8Gipec8Eeeu0xXdbba9frFj0=OqFfea0dXdd9vqai=hGuQ8kuc9pgc9s8qqaq=dirpe0xb9q8qiLsFr0=vr0=vr0dc8meaabaqaciaacaGaaeqabaqabeGadaaakeaadaWadaabaeqabaGaeCyyae2aaWbaaSqabeaacqWH4baEaaaakeaacqWHHbqydaahaaWcbeqaaiabhogaJbaaaaGccaGLBbGaayzxaaGaeCypa0ZaamWaaqaabeqaaiabhMfaznaaCaaaleqabaGaeCiEaGhaaaGcbaGaeCywaK1aaWbaaSqabeaacqWHJbWyaaaaaOGaay5waiaaw2faaiabgwSixlabhkgaIjaaxMaacaWLjaWaaeWaaeaacqaI5aqoaiaawIcacaGLPaaaaaa@44A5@

Where

ax=Δx+μ⋅∫titi+1xdt
 MathType@MTEF@5@5@+=feaafiart1ev1aaatCvAUfKttLearuWrP9MDH5MBPbIqV92AaeXatLxBI9gBaebbnrfifHhDYfgasaacH8akY=wiFfYdH8Gipec8Eeeu0xXdbba9frFj0=OqFfea0dXdd9vqai=hGuQ8kuc9pgc9s8qqaq=dirpe0xb9q8qiLsFr0=vr0=vr0dc8meaabaqaciaacaGaaeqabaqabeGadaaakeaacqWHHbqydaahaaWcbeqaaiabhIha4baakiabg2da9iabfs5aejabhIha4jabgUcaRGGaciab=X7aTjabgwSixpaapedabaGaeCiEaGNaemizaqMaemiDaqhaleaacqWG0baDdaWgaaadbaGaemyAaKgabeaaaSqaaiabdsha0naaBaaameaacqWGPbqAcqGHRaWkcqaIXaqmaeqaaaqdcqGHRiI8aaaa@46B8@

ac=Δc−(D⋅∫titi+1(cfeed−c)dt+Sc⋅J0⋅i⋅cX⋅Δt)
 MathType@MTEF@5@5@+=feaafiart1ev1aaatCvAUfKttLearuWrP9MDH5MBPbIqV92AaeXatLxBI9gBaebbnrfifHhDYfgasaacH8akY=wiFfYdH8Gipec8Eeeu0xXdbba9frFj0=OqFfea0dXdd9vqai=hGuQ8kuc9pgc9s8qqaq=dirpe0xb9q8qiLsFr0=vr0=vr0dc8meaabaqaciaacaGaaeqabaqabeGadaaakeaacqWHHbqydaahaaWcbeqaaiabhogaJbaakiabg2da9iabfs5aejabhogaJjabgkHiTmaabmaabaGaemiraqKaeyyXIC9aa8qmaeaadaqadaqaaiabhogaJnaaBaaaleaacqWGMbGzcqWGLbqzcqWGLbqzcqWGKbazaeqaaOGaeyOeI0IaeC4yamgacaGLOaGaayzkaaGaemizaqMaemiDaqhaleaacqWG0baDdaWgaaadbaGaemyAaKgabeaaaSqaaiabdsha0naaBaaameaacqWGPbqAcqGHRaWkcqaIXaqmaeqaaaqdcqGHRiI8aOGaey4kaSIaeC4uam1aaSbaaSqaaiabdogaJbqabaGccqGHflY1cqWHkbGsdaahaaWcbeqaaiabhcdaWaaakiabgwSixlabhMgaPjabgwSixlabdogaJnaaBaaaleaacqWGybawaeqaaOGaeyyXICTaeuiLdqKaemiDaqhacaGLOaGaayzkaaaaaa@6672@

and **Y **is a ((*q *- 1)·(*m*_*x *_+ *m*_*c*_)) × *p *matrix, combining **Y**^**x **^and **Y**^**c**^, containing the time integrals of the logarithm of the normalized metabolite concentrations, and **S**, **S**_**c**_, **J**^**0**^. Here *q *is the number of measured time points. **b **is a (*p *× 1) vector containing the unknown elasticity coefficients, which is estimated by linear regression similarly as above in eq. (5). To obtain the integrals of equation(9), a linear interpolation of concentration between the measurements at t_i _and t_i+1 _is used.

Although both equations ((6) and (5)) are based on least square principle, the use of equation (6) (where **b **of equation (5) is used as an initial estimate) has advantages, namely, further improvement of the quality of the estimated parameters, because initial linear regression assumes that errors are only present in the dependent variables (**a **in Eq. (5)), whereas errors in the measured metabolites in fact also affect the independent variables (matrix **Y **in Eq.(4) and Eq.(9)). Furthermore, the non-linear optimization allows the incorporation of additional degrees of freedom for the correction of errors in the metabolite levels at the first data point (t_0_) used for the model simulation (integration of Eq.(8)). Moreover, during the non-linear parameter estimation, linear interpolation between the logarithms of the measured metabolites is not needed anymore. An additional ease of the non-linear regression is in the introduction of the adjustable parameter *γ *to weigh the effect of one (or more) metabolite(s) on the fit. This is useful, when the measurement precision is poor for a certain metabolite or when the relative change in one metabolite is small compared to the others. The obvious choice for *γ *would be the inverse of the standard deviation of the corresponding metabolite. Although the weighing parameter *γ *can be introduced in the linear regression step as well, introducing the factor *γ *in the non-linear regression step is much more straightforward.

### Calculation of the systemic properties

Having estimated the elasticities, we can calculate scaled flux and concentration control coefficients (CvkJ=vk0J⋅∂J∂vk|0,Cvkxi=vk0xi0⋅∂xi∂vk|0)
 MathType@MTEF@5@5@+=feaafiart1ev1aqatCvAUfKttLearuWrP9MDH5MBPbIqV92AaeXatLxBI9gBaebbnrfifHhDYfgasaacH8akY=wiFfYdH8Gipec8Eeeu0xXdbba9frFj0=OqFfea0dXdd9vqai=hGuQ8kuc9pgc9s8qqaq=dirpe0xb9q8qiLsFr0=vr0=vr0dc8meaabaqaciaacaGaaeqabaqabeGadaaakeaadaqadaqaaiabdoeadnaaDaaaleaacqWG2bGDdaWgaaadbaGaem4AaSgabeaaaSqaaiabdQeakbaakiabg2da9maalaaabaGaemODay3aa0baaSqaaiabdUgaRbqaaiabicdaWaaaaOqaaiabdQeakbaacqGHflY1daabcaqaamaalaaabaGaeyOaIyRaemOsaOeabaGaeyOaIyRaemODay3aaSbaaSqaaiabdUgaRbqabaaaaaGccaGLiWoadaWgaaWcbaGaeGimaadabeaakiabcYcaSiabdoeadnaaDaaaleaacqWG2bGDdaWgaaadbaGaem4AaSgabeaaaSqaaiabdIha4naaBaaameaacqWGPbqAaeqaaaaakiabg2da9maalaaabaGaemODay3aa0baaSqaaiabdUgaRbqaaiabicdaWaaaaOqaaiabdIha4naaDaaaleaacqWGPbqAaeaacqaIWaamaaaaaOGaeyyXIC9aaqGaaeaadaWcaaqaaiabgkGi2kabdIha4naaBaaaleaacqWGPbqAaeqaaaGcbaGaeyOaIyRaemODay3aaSbaaSqaaiabdUgaRbqabaaaaaGccaGLiWoadaWgaaWcbaGaeGimaadabeaaaOGaayjkaiaawMcaaaaa@6507@, using classical summation and connectivity theorems [34]:

**C**^**x0 **^= **-L**^**x**^·(**S**·**J**^**0**^·**E**^**x0**^·**L**^**x**^)^**-1**^·**S**·**J**^**0 **^    (10)

**C**^**J0 **^= **I **+ **E**^**x0**^·**C**^**x0 **^    (11)

Here **L**^**x **^is the metabolite link matrix, which links the dependent metabolites to the independent ones and **J**^**0**^, **S **and **E**^**x0 **^are steady state flux matrix, stoichiometric matrix and the elasticity matrix as represented in the previous sections. The scaled response coefficient (**R**^**J0**^) for the external metabolites can also be calculated [43]:

**R**^**J0 **^= **C**^**J0**^·**E**^**c0 **^    (12)

### Metabolic model

The glycolytic pathway of *Saccharomyces cerevisiae *has been extensively studied in terms of enzyme kinetics and metabolite levels. Over the last 30 years, modeling of the glycolysis of yeast cells has been applied for several reasons, such as simulation of physiology to understand regulation under dynamic conditions, MCA to amplify and/or redirect metabolic flux [29,31,44-47], and to investigate how changing environmental conditions change metabolism.

**Figure 10:**
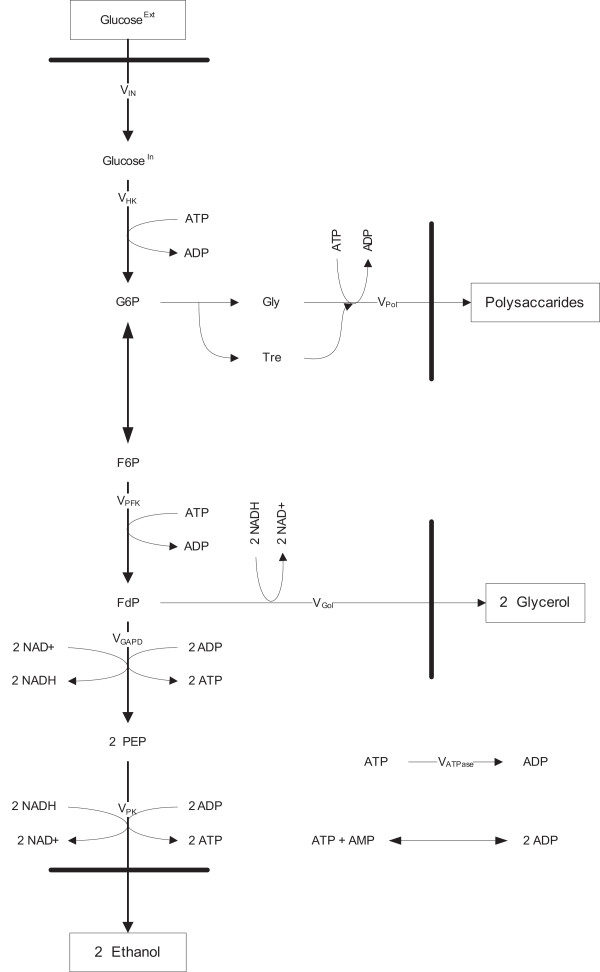
Glycolytic Pathway of *Saccharomyces cerevisiae *(Adapted from Galazzo and Bailey, 1990).

In order to test the proposed method for estimation of elasticities from steady state and dynamic perturbations and the applicability of linlog kinetics, noise-free data were generated using the modified version of the mechanistic model of Galazzo and Bailey describing the glycolysis of yeast cells, which contains 8 reactions and 9 metabolites (Figure 10). The set of mechanistic kinetic equations are highly non-linear and contain 41 parameters. The parameters used were taken from Galazzo and Bailey [31] using the experimental settings for pH^ext ^= 5.5 with suspended cells in the original study. The glucose uptake rate of the original model was modified in order to be able to mimic realistic fermentation conditions. The details of the metabolic model, the list of mechanistic rate equations and the mass balances describing the kinetics of the metabolic network of Figure 10 are given in the Appendix.

### Reactor model

The chosen glycolytic model was not designed to describe growth, so in order to prevent washout of the cells and to keep a constant amount of biomass within the fermentor, the organism was assumed to be cultivated in a chemostat with complete biomass retention. The glucose concentration in the feed was set at a low value resulting in low steady state ethanol and glycerol concentrations, thus allowing measurable changes in those metabolites when a perturbation was applied. Furthermore the biomass concentration was set at 5 gDW L^-1 ^and the dilution rate was set at 1.5 hr^-1 ^to provide a reasonable substrate load (q_s_) and glycolytic flux. The resulting reference steady state conditions (fluxes, metabolite levels) are represented in Table 3.

**Table 3:** Reference conditions of fermentation and microorganism

**Fermentation parameters**	
D = 1.5 hr^-1^, cGlucosefeed MathType@MTEF@5@5@+=feaafiart1ev1aaatCvAUfKttLearuWrP9MDH5MBPbIqV92AaeXatLxBI9gBaebbnrfifHhDYfgasaacH8akY=wiFfYdH8Gipec8Eeeu0xXdbba9frFj0=OqFfea0dXdd9vqai=hGuQ8kuc9pgc9s8qqaq=dirpe0xb9q8qiLsFr0=vr0=vr0dc8meaabaqaciaacaGaaeqabaqabeGadaaakeaacqWGJbWydaqhaaWcbaGaee4raCKaeeiBaWMaeeyDauNaee4yamMaee4Ba8Maee4CamNaeeyzaugabaGaemOzayMaemyzauMaemyzauMaemizaqgaaaaa@3CC9@ = 8000 *μ*mol L^-1^, *c*_*X *_= 5 gDW L^-1^	

**Biological parameters**	

**Extracellular metabolite concentrations **[*μ*mol L^-1^]	

G^ext^	2666.8
Pol	72.7
Gol	664.5
EtOH	9856.5
**Macroscopic Fluxes **[*μ*mol gDW^-1 ^hr^-1^]	

q_s_	1600
q_Pol_	21.8
q_Gol_	199.4
q_EtOH_	2957
**Intracellular metabolite concentrations **[*μ*mol gDW^-1^]	

G^in^	0.0524
G6P	3.133
FdP	11.657
PEP	0.0149
ATP	3.738
**Intracellular rates **[*μ*mol gDW^-1 ^hr^-1^]	

V_IN_	1600
V_HK_	1600
V_PFK_	1578.2
V_GAPD_	1478.5
V_PK_	2957
V_Pol_	21.8
V_Gol_	199.4
V_ATPase_	2714

### Linlog kinetic model

**Figure 11:**
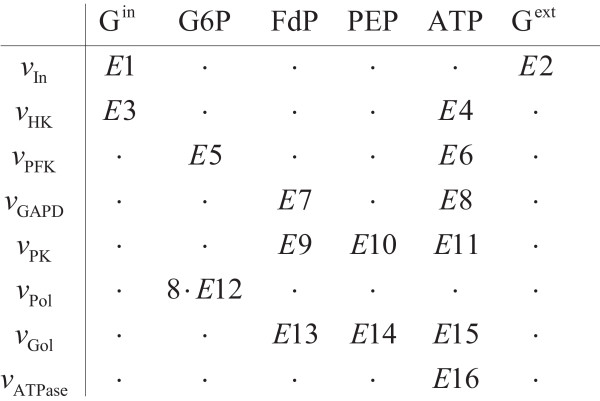
Nonzero entries of the elasticity matrix. Adapted from Galazzo and Bailey, 1990, with modifications in the V_IN _reaction. For the modifications in the uptake reaction, see text.

The number of non-zero entries of the elasticity matrix ([**E**^**x **^**E**^**c**^] in equation(3)) defines the number of elasticities to be estimated. Specifically, the elasticity matrix has 72 entries (8 reactions × 9 metabolites) and it followed that 16 of these were nonzero for the Galazzo and Bailey model which was extended with the alterations on the uptake reaction, mentioned in the Appendix. For the present network, the structure of the elasticity matrix is presented in Figure 11. The linlog model contains 16 elasticities and 8 reference rates as parameters whereas the mechanistic model has 41 parameters.

### Dynamic perturbations

To obtain transient data, the reference steady state (Table 3) was perturbed by increasing the extracellular glucose concentration (G^ext^) at t = 0 from 2666 *μ*mol L^-1 ^to 8000 *μ*mol L^-1^, where after the relaxation of the system was simulated. The levels of the metabolites were recorded until the system returned to the reference state.

In choosing the number of data points, i.e. the sampling frequency, special attention had been paid to use a realistic number of data points that an experimenter can obtain from a typical rapid sampling experiment (for a short time period) or from a dynamic pulse experiment (for a longer time period). The data were obtained as follows: in the first 120 seconds, where the initial dynamics after the glucose uptake are important, 20 samples uniformly distributed over time were taken, in the next 120 seconds, 10 samples and in the last minute 5 samples were taken. For long term experiments, from the 5^th ^minute until the 10^th ^minute, 5 samples were taken, and from the 10^th ^minute until the end (400 min) 30 uniformly distributed samples were taken. Metabolites for which concentrations were assumed to be available at these times are intracellular glucose, G6P, FdP, PEP, ATP and extracellular metabolites glucose, ethanol, glycerol and polysaccharides.

### Steady state perturbations

For the steady state perturbations, the enzyme levels were modulated and the system was allowed to reach a new steady state. The new steady state conditions (fluxes, metabolite levels) were measured.

The minimum number of independent steady state perturbations needed in order to identify all the elasticities for a reaction equals the number of non-zero elasticities for that rate expression. From Figure 11, where the elasticity matrix is shown, it can be inferred that the minimum number of perturbations to be applied per reaction is 3, due to the V_PK _and V_Gol _reactions that have three non-zero elasticities (E9–E10–E11 and E13–E14–E15 respectively).

As the reaction to be perturbed, V_IN _was chosen because it is at the beginning of the glycolysis and therefore affects the entire pathway. As the second reaction to be perturbed, V_ATPase _was chosen because it directly affects the level of ATP which is the effector of 6 out of 8 reactions. Lastly, V_GAPD _was chosen for two reasons: 1) to resolve the branch point relations around G6P and 2) to resolve the feed forward effect of FdP on V_PK_. Note that since the production rate of glycerol (V_Gol_) was coupled (proportional) to the production of ethanol (V_PK_), the steady state branch point relations around FdP can be reformulated as follows:

At steady state, *J*_*PFK *_= *J*_*GAPD *_+ *J*_*Gol *_and *J*_*GAPD *_= *J*_*PK*_/2. Additionally, as described above, *J*_*Gol *_= *k*·*J*_*PK*_, hence *J*_*PFK *_= (1 + 2·*k*)·*J*_*GAPD *_where the value of *k *is given in the Appendix. Therefore, there is no need to introduce additional steady state perturbations to resolve the branch point relations around FdP. Table 1 summarizes the steady state perturbations used as data in this study. For example, in the first column, the activity of the glucose uptake rate was increased by 20% and the system is allowed to reach the new steady state, the fluxes and the metabolite levels relative to the reference state are presented in the corresponding column. Similarly, the second column represents the inhibition of V_ATPase _by 10% and the third represents the data after the inhibition of V_GAPD _by 20%.

### Error propagation analysis

In order to inspect whether the estimated elasticities are robust against experimental noise in metabolite concentrations, we performed Monte Carlo (MC) simulations. To this end, random errors of 10% are repeatedly added to form 50 sets of "noisy data", which were used in the non-linear parameter estimation procedure. This yields a distribution of estimated elasticities which allows calculating the relative standard deviation for each elasticity as the ratio of standard deviation to the mean of that elasticity distribution.

### Parameter identifiability and model reduction

The elasticities that have a high relative standard deviation are suspected to be poorly identifiable in the network. Potentially unidentifiable elasticities were recognized by calculating a p-value for testing the null hypothesis that the true mean of a given elasticity distribution is zero through the statistic *μ*/*(σ*/N
 MathType@MTEF@5@5@+=feaafiart1ev1aaatCvAUfKttLearuWrP9MDH5MBPbIqV92AaeXatLxBI9gBaebbnrfifHhDYfgasaacH8akY=wiFfYdH8Gipec8Eeeu0xXdbba9frFj0=OqFfea0dXdd9vqai=hGuQ8kuc9pgc9s8qqaq=dirpe0xb9q8qiLsFr0=vr0=vr0dc8meaabaqaciaacaGaaeqabaqabeGadaaakeaadaGcaaqaaiabd6eaobWcbeaaaaa@2DEC@) that has a t-distribution with N degrees of freedom, where N is the number of MC simulations. The elasticities that were found to be potentially unidentifiable were set to zero, thus reducing the number of non-zero entries in the elasticity matrix. Subsequently, for the reduced model, the remaining elasticities were re-estimated, using the same steady state and dynamic perturbation data and the parameter estimation procedure explained in the previous sections.

### Software used

MATLAB software (version 6.5, Stat-Ease Inc., Minneapolis, USA) was used for generating the *in silico *data and analyses of the data and the parameters obtained. The non-linear minimization problem of parameter estimation was solved using the Nelder-Mead simplex search method.

## Authors' contributions

IEN set up the methodology, performed the calculations and drafted the manuscript. WvW supervised the calculations, the organization of the manuscript and revised the manuscript. WvG contributed in organizing and revising the manuscript. JJH supervised the whole project. All of the authors read and approved the final manuscript.

## Appendix

### Details of the in silico metabolic model

In this paper, the model of Galazzo and Bailey describing the glycolysis of yeast cells is used. This section describes the details of the metabolic model. The model consists of 5 intracellular (glucose^intracellular^, G6P, FdP, PEP and ATP), 4 extracellular (glucose^extracellular^, polysaccharides, glycerol and ethanol) metabolites and 8 reactions. In their work, a mathematical model for the description of ethanol production by non-growing *Saccharomyces cerevisiae *has been constructed using non-linear mechanistic rate equations. The glucose uptake is modeled by symmetrical carrier model (see below for the changes in the uptake reaction), the hexokinase reaction is described by a two substrate single displacement mechanism, the phosphofructokinase and pyruvate kinase reactions are described by allosteric kinetics according to Monod-Wyman-Changeux, polysaccharide formation is explained by Hill type kinetics and the glyceraldehyde 3-phosphate dehydrogenase reaction is described by a kinetic expression which takes into account the crossed product inhibition by G3P and NAD^+ ^and competitive inhibition by AMP, ADP and ATP. The ATP consumption is assumed to have first order kinetics.

One property of the original model is that the glycerol production is assumed to be stoichiometrically coupled to ethanol formation, i.e. there is no separate dynamics for the glycerol formation. Additionally, the NAD^+ ^and NADH levels are assumed to be constant, as given below.

As it was originally published, the glucose influx was a linear function of the glucose-6-phosphate concentration only; hence, it was insensitive to the changes in extracellular glucose concentration. To mimic realistic fermentation conditions, where one can perturb the organism by adding a specific compound such as glucose to a chemostat, we have modified the glucose uptake reaction and modeled the glucose uptake by a symmetrical carrier model, suggesting that the transport of glucose across the cell membrane occurs via facilitated diffusion, as proposed by Teusink et al., [4]. The list of mechanistic rate equations used is given in the following section.

### Mechanistic rate equations used to construct the in silico network. (Adapted from Galazzo and Bailey, 1990)

The rate equations of the reactions in Figure 10 are given by:

VIN=17.223(Gext−Gin)1.19181+Gext1.1918+Gin1.1918+0.91Gext⋅Gin1.1918⋅1.1918
 MathType@MTEF@5@5@+=feaafiart1ev1aaatCvAUfKttLearuWrP9MDH5MBPbIqV92AaeXatLxBI9gBaebbnrfifHhDYfgasaacH8akY=wiFfYdH8Gipec8Eeeu0xXdbba9frFj0=OqFfea0dXdd9vqai=hGuQ8kuc9pgc9s8qqaq=dirpe0xb9q8qiLsFr0=vr0=vr0dc8meaabaqaciaacaGaaeqabaqabeGadaaakeaacqWGwbGvdaWgaaWcbaGaemysaKKaemOta4eabeaakiabg2da9iabbgdaXiabbEda3iabb6caUiabbkdaYiabbkdaYiabbodaZmaalaaabaWaaSaaaeaadaqadaqaaiabdEeahnaaCaaaleqabaGaemyzauMaemiEaGNaemiDaqhaaOGaeyOeI0Iaem4raC0aaWbaaSqabeaacqWGPbqAcqWGUbGBaaaakiaawIcacaGLPaaaaeaacqqGXaqmcqqGUaGlcqqGXaqmcqqG5aqocqqGXaqmcqqG4aaoaaaabaGaeGymaeJaey4kaSYaaSaaaeaacqWGhbWrdaahaaWcbeqaaiabdwgaLjabdIha4jabdsha0baaaOqaaiabbgdaXiabb6caUiabbgdaXiabbMda5iabbgdaXiabbIda4aaacqGHRaWkdaWcaaqaaiabdEeahnaaCaaaleqabaGaemyAaKMaemOBa4gaaaGcbaGaeeymaeJaeeOla4IaeeymaeJaeeyoaKJaeeymaeJaeeioaGdaaiabgUcaRiabicdaWiabc6caUiabiMda5iabigdaXmaalaaabaGaem4raC0aaWbaaSqabeaacqWGLbqzcqWG4baEcqWG0baDaaGccqGHflY1cqWGhbWrdaahaaWcbeqaaiabdMgaPjabd6gaUbaaaOqaaiabbgdaXiabb6caUiabbgdaXiabbMda5iabbgdaXiabbIda4iabgwSixlabbgdaXiabb6caUiabbgdaXiabbMda5iabbgdaXiabbIda4aaaaaaaaa@7E24@

VHK=68.5⋅(10.0062⋅0.1Gin⋅ATP+0.11Gin+0.1ATP+1)
 MathType@MTEF@5@5@+=feaafiart1ev1aaatCvAUfKttLearuWrP9MDH5MBPbIqV92AaeXatLxBI9gBaebbnrfifHhDYfgasaacH8akY=wiFfYdH8Gipec8Eeeu0xXdbba9frFj0=OqFfea0dXdd9vqai=hGuQ8kuc9pgc9s8qqaq=dirpe0xb9q8qiLsFr0=vr0=vr0dc8meaabaqaciaacaGaaeqabaqabeGadaaakeaacqWGwbGvdaWgaaWcbaGaemisaGKaem4saSeabeaakiabg2da9iabiAda2iabiIda4iabc6caUiabiwda1iabgwSixpaabmaabaWaaSaaaeaacqaIXaqmaeaadaWcaaqaaiabicdaWiabc6caUiabicdaWiabicdaWiabiAda2iabikdaYiabgwSixlabicdaWiabc6caUiabigdaXaqaaiabdEeahnaaCaaaleqabaGaemyAaKMaemOBa4gaaOGaeyyXICTaemyqaeKaemivaqLaemiuaafaaiabgUcaRmaalaaabaGaeGimaaJaeiOla4IaeGymaeJaeGymaedabaGaem4raC0aaWbaaSqabeaacqWGPbqAcqWGUbGBaaaaaOGaey4kaSYaaSaaaeaacqaIWaamcqGGUaGlcqaIXaqmaeaacqWGbbqqcqWGubavcqWGqbauaaGaey4kaSIaeGymaedaaaGaayjkaiaawMcaaaaa@600C@

VPFK=31.7166.67⋅F6P⋅ATP⋅RR2+LT2
 MathType@MTEF@5@5@+=feaafiart1ev1aaatCvAUfKttLearuWrP9MDH5MBPbIqV92AaeXatLxBI9gBaebbnrfifHhDYfgasaacH8akY=wiFfYdH8Gipec8Eeeu0xXdbba9frFj0=OqFfea0dXdd9vqai=hGuQ8kuc9pgc9s8qqaq=dirpe0xb9q8qiLsFr0=vr0=vr0dc8meaabaqaciaacaGaaeqabaqabeGadaaakeaacqWGwbGvdaWgaaWcbaGaemiuaaLaemOrayKaem4saSeabeaakiabg2da9iabiodaZiabigdaXiabc6caUiabiEda3maalaaabaGaeGymaeJaeGOnayJaeGOnayJaeiOla4IaeGOnayJaeG4naCJaeyyXICTaemOrayKaeGOnayJaemiuaaLaeyyXICTaemyqaeKaemivaqLaemiuaaLaeyyXICTaemOuaifabaGaemOuai1aaWbaaSqabeaacqaIYaGmaaGccqGHRaWkcqWGmbatcqWGubavdaahaaWcbeqaaiabikdaYaaaaaaaaa@515D@

with

*R *= 1 + *F*6*P *+ 16.67 *ATP *+ 16.67*F*6*P*·*ATP*

*T *= 1 + 5.10^-4 ^*F*6*P *+ 16.67 *ATP *+ 0.00833*F*6*P*·*ATP*

L=L0(1+0.76AMP1+40AMP)2
 MathType@MTEF@5@5@+=feaafiart1ev1aaatCvAUfKttLearuWrP9MDH5MBPbIqV92AaeXatLxBI9gBaebbnrfifHhDYfgasaacH8akY=wiFfYdH8Gipec8Eeeu0xXdbba9frFj0=OqFfea0dXdd9vqai=hGuQ8kuc9pgc9s8qqaq=dirpe0xb9q8qiLsFr0=vr0=vr0dc8meaabaqaciaacaGaaeqabaqabeGadaaakeaacqWGmbatcqGH9aqpcqWGmbatdaWgaaWcbaGaeGimaadabeaakmaabmaabaWaaSaaaeaacqaIXaqmcqGHRaWkcqaIWaamcqGGUaGlcqaI3aWncqaI2aGncqWGbbqqcqWGnbqtcqWGqbauaeaacqaIXaqmcqGHRaWkcqaI0aancqaIWaamcqWGbbqqcqWGnbqtcqWGqbauaaaacaGLOaGaayzkaaWaaWbaaSqabeaacqaIYaGmaaaaaa@43CE@, L_0 _is pH dependent

VGAPD=49.9[1+0.0025G3P+0.18NAD+(1+∑i=13ζiKi)+0.0025⋅0.18G3P⋅NAD+(1+NADH0.0003)(1+∑i=13ζiKi)]−1
 MathType@MTEF@5@5@+=feaafiart1ev1aaatCvAUfKttLearuWrP9MDH5MBPbIqV92AaeXatLxBI9gBaebbnrfifHhDYfgasaacH8akY=wiFfYdH8Gipec8Eeeu0xXdbba9frFj0=OqFfea0dXdd9vqai=hGuQ8kuc9pgc9s8qqaq=dirpe0xb9q8qiLsFr0=vr0=vr0dc8meaabaqaciaacaGaaeqabaqabeGadaaakeaacqWGwbGvdaWgaaWcbaGaem4raCKaemyqaeKaemiuaaLaemiraqeabeaakiabg2da9iabisda0iabiMda5iabc6caUiabiMda5maadmaabaGaeGymaeJaey4kaSYaaSaaaeaacqaIWaamcqGGUaGlcqaIWaamcqaIWaamcqaIYaGmcqaI1aqnaeaacqWGhbWrcqaIZaWmcqWGqbauaaGaey4kaSYaaSaaaeaacqaIWaamcqGGUaGlcqaIXaqmcqaI4aaoaeaacqWGobGtcqWGbbqqcqWGebarcqGHRaWkaaWaaeWaaeaacqaIXaqmcqGHRaWkdaaeWbqaamaalaaabaacciGae8NTdO3aaSbaaSqaaiabdMgaPbqabaaakeaacqWGlbWsdaWgaaWcbaGaemyAaKgabeaaaaaabaGaemyAaKMaeyypa0JaeGymaedabaGaeG4mamdaniabggHiLdaakiaawIcacaGLPaaacqGHRaWkdaWcaaqaaiabicdaWiabc6caUiabicdaWiabicdaWiabikdaYiabiwda1iabgwSixlabicdaWiabc6caUiabigdaXiabiIda4aqaaiabdEeahjabiodaZiabdcfaqjabgwSixlabd6eaojabdgeabjabdseaejabgUcaRaaadaqadaqaaiabigdaXiabgUcaRmaalaaabaGaemOta4KaemyqaeKaemiraqKaemisaGeabaGaeGimaaJaeiOla4IaeGimaaJaeGimaaJaeGimaaJaeG4mamdaaaGaayjkaiaawMcaamaabmaabaGaeGymaeJaey4kaSYaaabCaeaadaWcaaqaaiab=z7a6naaBaaaleaacqWGPbqAaeqaaaGcbaGaem4saS0aaSbaaSqaaiabdMgaPbqabaaaaaqaaiabdMgaPjabg2da9iabigdaXaqaaiabiodaZaqdcqGHris5aaGccaGLOaGaayzkaaaacaGLBbGaayzxaaWaaWbaaSqabeaacqGHsislcqaIXaqmaaaaaa@9228@

with

*ζ*_*i*,*i*=1,2,3 _= *AMP*, *ADP*, *ATP *    *K*_*i*,*i*=1,2,3 _= 1.1, 1.5, 2.5

VPK=(34401+KpHH+)3.1976⋅PEP⋅ADP⋅R+0.0004PEP⋅ADP⋅T⋅LR2+LT2
 MathType@MTEF@5@5@+=feaafiart1ev1aaatCvAUfKttLearuWrP9MDH5MBPbIqV92AaeXatLxBI9gBaebbnrfifHhDYfgasaacH8akY=wiFfYdH8Gipec8Eeeu0xXdbba9frFj0=OqFfea0dXdd9vqai=hGuQ8kuc9pgc9s8qqaq=dirpe0xb9q8qiLsFr0=vr0=vr0dc8meaabaqaciaacaGaaeqabaqabeGadaaakeaacqWGwbGvdaWgaaWcbaGaemiuaaLaem4saSeabeaakiabg2da9maabmaabaWaaSaaaeaacqaIZaWmcqaI0aancqaI0aancqaIWaamaeaacqaIXaqmcqGHRaWkdaWccaqaaiabdUealnaaBaaaleaacqWGWbaCcqWGibasaeqaaaGcbaGaemisaGKaey4kaScaaaaaaiaawIcacaGLPaaadaWcaaqaaiabiodaZiabc6caUiabigdaXiabiMda5iabiEda3iabiAda2iabgwSixlabdcfaqjabdweafjabdcfaqjabgwSixlabdgeabjabdseaejabdcfaqjabgwSixlabdkfasjabgUcaRiabicdaWiabc6caUiabicdaWiabicdaWiabicdaWiabisda0iabdcfaqjabdweafjabdcfaqjabgwSixlabdgeabjabdseaejabdcfaqjabgwSixlabdsfaujabgwSixlabdYeambqaaiabdkfasnaaCaaaleqabaGaeGOmaidaaOGaey4kaSIaemitaWKaemivaq1aaWbaaSqabeaacqaIYaGmaaaaaaaa@6FFC@

with

*R *= 1 + 159.88*PEP *+ 0.2*ADP *+ 3.1976*PEP*·*ADP*

*T *= 1 + 0.02*PEP *+ 0.2*ADP *+ 0.004*PEP*·*ADP*

L=L0⋅(1+0.05⋅FdP1+5⋅FdP)2
 MathType@MTEF@5@5@+=feaafiart1ev1aaatCvAUfKttLearuWrP9MDH5MBPbIqV92AaeXatLxBI9gBaebbnrfifHhDYfgasaacH8akY=wiFfYdH8Gipec8Eeeu0xXdbba9frFj0=OqFfea0dXdd9vqai=hGuQ8kuc9pgc9s8qqaq=dirpe0xb9q8qiLsFr0=vr0=vr0dc8meaabaqaciaacaGaaeqabaqabeGadaaakeaacqWGmbatcqGH9aqpcqWGmbatdaWgaaWcbaGaeGimaadabeaakiabgwSixpaabmaabaWaaSaaaeaacqaIXaqmcqGHRaWkcqaIWaamcqGGUaGlcqaIWaamcqaI1aqncqGHflY1cqWGgbGrcqWGKbazcqWGqbauaeaacqaIXaqmcqGHRaWkcqaI1aqncqGHflY1cqWGgbGrcqWGKbazcqWGqbauaaaacaGLOaGaayzkaaWaaWbaaSqabeaacqaIYaGmaaaaaa@4A20@, L_0 _pH dependent

VPol=1.1⋅VGlyVGlyapp=14.31⋅G6P8.2528.25+G6P8.25⋅(11UDPG(1+1.1G6P)+1)
 MathType@MTEF@5@5@+=feaafiart1ev1aaatCvAUfKttLearuWrP9MDH5MBPbIqV92AaeXatLxBI9gBaebbnrfifHhDYfgasaacH8akY=wiFfYdH8Gipec8Eeeu0xXdbba9frFj0=OqFfea0dXdd9vqai=hGuQ8kuc9pgc9s8qqaq=dirpe0xb9q8qiLsFr0=vr0=vr0dc8meaabaqaciaacaGaaeqabaqabeGadaaakeaacqWGwbGvdaWgaaWcbaGaemiuaaLaem4Ba8MaemiBaWgabeaakiabg2da9iabigdaXiabc6caUiabigdaXiabgwSixlabdAfawnaaBaaaleaacqWGhbWrcqWGSbaBcqWG5bqEaeqaaOGaemOvay1aa0baaSqaaiabdEeahjabdYgaSjabdMha5bqaaiabdggaHjabdchaWjabdchaWbaakiabg2da9maalaaabaGaeGymaeJaeGinaqJaeiOla4IaeG4mamJaeGymaeJaeyyXICTaem4raCKaeGOnayJaemiuaa1aaWbaaSqabeaacqaI4aaocqGGUaGlcqaIYaGmcqaI1aqnaaaakeaacqaIYaGmdaahaaWcbeqaaiabiIda4iabc6caUiabikdaYiabiwda1aaakiabgUcaRiabdEeahjabiAda2iabdcfaqnaaCaaaleqabaGaeGioaGJaeiOla4IaeGOmaiJaeGynaudaaaaakiabgwSixpaabmaabaWaaSaaaeaacqaIXaqmaeaadaWcaaqaaiabigdaXaqaaiabdwfavjabdseaejabdcfaqjabdEeahbaadaqadaqaaiabigdaXiabgUcaRmaalaaabaGaeGymaeJaeiOla4IaeGymaedabaGaem4raCKaeGOnayJaemiuaafaaaGaayjkaiaawMcaaiabgUcaRiabigdaXaaaaiaawIcacaGLPaaaaaa@78E7@

*V*_*Gol *_= 0.06742·*V*_*PK*_

*V*_*ATPase *_= 12.1·*ATP*

### Equilibrium relations

The following metabolites were assumed in equilibrium throughout the simulated time window:

ATP+AMP↔AK2ADPADP2ATP⋅AMP=1
 MathType@MTEF@5@5@+=feaafiart1ev1aaatCvAUfKttLearuWrP9MDH5MBPbIqV92AaeXatLxBI9gBaebbnrfifHhDYfgasaacH8akY=wiFfYdH8Gipec8Eeeu0xXdbba9frFj0=OqFfea0dXdd9vqai=hGuQ8kuc9pgc9s8qqaq=dirpe0xb9q8qiLsFr0=vr0=vr0dc8meaabaqaciaacaGaaeqabaqabeGadaaakeaafaqabeqacaaabaGaemyqaeKaemivaqLaemiuaaLaey4kaSIaemyqaeKaemyta0Kaemiuaa1aa4anaSqaaiabbgeabjabbUealbqabOGaayjLHaGaeGOmaiJaemyqaeKaemiraqKaemiuaafabaWaaSaaaeaacqWGbbqqcqWGebarcqWGqbaudaahaaWcbeqaaiabikdaYaaaaOqaaiabdgeabjabdsfaujabdcfaqjabgwSixlabdgeabjabd2eanjabdcfaqbaacqGH9aqpcqaIXaqmaaaaaa@4BD8@

G6P↔F6PF6PG6P=0.3
 MathType@MTEF@5@5@+=feaafiart1ev1aaatCvAUfKttLearuWrP9MDH5MBPbIqV92AaeXatLxBI9gBaebbnrfifHhDYfgasaacH8akY=wiFfYdH8Gipec8Eeeu0xXdbba9frFj0=OqFfea0dXdd9vqai=hGuQ8kuc9pgc9s8qqaq=dirpe0xb9q8qiLsFr0=vr0=vr0dc8meaabaqaciaacaGaaeqabaqabeGadaaakeaafaqabeqacaaabaGaem4raCKaeGOnayJaemiuaaLaeyiLHSQaemOrayKaeGOnayJaemiuaafabaWaaSaaaeaacqWGgbGrcqaI2aGncqWGqbauaeaacqWGhbWrcqaI2aGncqWGqbauaaGaeyypa0JaeGimaaJaeiOla4IaeG4mamdaaaaa@3F65@

FdP↔G3PG3PFdP=0.01
 MathType@MTEF@5@5@+=feaafiart1ev1aaatCvAUfKttLearuWrP9MDH5MBPbIqV92AaeXatLxBI9gBaebbnrfifHhDYfgasaacH8akY=wiFfYdH8Gipec8Eeeu0xXdbba9frFj0=OqFfea0dXdd9vqai=hGuQ8kuc9pgc9s8qqaq=dirpe0xb9q8qiLsFr0=vr0=vr0dc8meaabaqaciaacaGaaeqabaqabeGadaaakeaafaqabeqacaaabaGaemOrayKaemizaqMaemiuaaLaeyiLHSQaem4raCKaeG4mamJaemiuaafabaWaaSaaaeaacqWGhbWrcqaIZaWmcqWGqbauaeaacqWGgbGrcqWGKbazcqWGqbauaaGaeyypa0JaeGimaaJaeiOla4IaeGimaaJaeGymaedaaaaa@40F1@

3PG↔PEPPEP3PG=0.1
 MathType@MTEF@5@5@+=feaafiart1ev1aaatCvAUfKttLearuWrP9MDH5MBPbIqV92AaeXatLxBI9gBaebbnrfifHhDYfgasaacH8akY=wiFfYdH8Gipec8Eeeu0xXdbba9frFj0=OqFfea0dXdd9vqai=hGuQ8kuc9pgc9s8qqaq=dirpe0xb9q8qiLsFr0=vr0=vr0dc8meaabaqaciaacaGaaeqabaqabeGadaaakeaafaqabeqacaaabaGaeG4mamJaemiuaaLaem4raCKaeyiLHSQaemiuaaLaemyrauKaemiuaafabaWaaSaaaeaacqWGqbaucqWGfbqrcqWGqbauaeaacqaIZaWmcqWGqbaucqWGhbWraaGaeyypa0JaeGimaaJaeiOla4IaeGymaedaaaaa@3FAF@

### Mass balances

The concentrations of the extracellular metabolites are described by the following mass balances:

dcGextdt=D⋅(cGextfeed−cGext)−VIN⋅cX
 MathType@MTEF@5@5@+=feaafiart1ev1aaatCvAUfKttLearuWrP9MDH5MBPbIqV92AaeXatLxBI9gBaebbnrfifHhDYfgasaacH8akY=wiFfYdH8Gipec8Eeeu0xXdbba9frFj0=OqFfea0dXdd9vqai=hGuQ8kuc9pgc9s8qqaq=dirpe0xb9q8qiLsFr0=vr0=vr0dc8meaabaqaciaacaGaaeqabaqabeGadaaakeaadaWcaaqaaiabdsgaKjabdogaJnaaBaaaleaacqWGhbWrdaahaaadbeqaaiabdwgaLjabdIha4jabdsha0baaaSqabaaakeaacqWGKbazcqWG0baDaaGaeyypa0JaemiraqKaeyyXIC9aaeWaaeaacqWGJbWydaqhaaWcbaGaem4raC0aaWbaaWqabeaacqWGLbqzcqWG4baEcqWG0baDaaaaleaacqWGMbGzcqWGLbqzcqWGLbqzcqWGKbazaaGccqGHsislcqWGJbWydaWgaaWcbaGaem4raC0aaWbaaWqabeaacqWGLbqzcqWG4baEcqWG0baDaaaaleqaaaGccaGLOaGaayzkaaGaeyOeI0IaemOvay1aaSbaaSqaaiabdMeajjabd6eaobqabaGccqGHflY1cqWGJbWydaWgaaWcbaGaemiwaGfabeaaaaa@5BBF@

dcPoldt=D⋅(cPolfeed−cPol)+VPol⋅cX
 MathType@MTEF@5@5@+=feaafiart1ev1aaatCvAUfKttLearuWrP9MDH5MBPbIqV92AaeXatLxBI9gBaebbnrfifHhDYfgasaacH8akY=wiFfYdH8Gipec8Eeeu0xXdbba9frFj0=OqFfea0dXdd9vqai=hGuQ8kuc9pgc9s8qqaq=dirpe0xb9q8qiLsFr0=vr0=vr0dc8meaabaqaciaacaGaaeqabaqabeGadaaakeaadaWcaaqaaiabdsgaKjabdogaJnaaBaaaleaacqWGqbaucqWGVbWBcqWGSbaBaeqaaaGcbaGaemizaqMaemiDaqhaaiabg2da9iabdseaejabgwSixpaabmaabaGaem4yam2aa0baaSqaaiabdcfaqjabd+gaVjabdYgaSbqaaiabdAgaMjabdwgaLjabdwgaLjabdsgaKbaakiabgkHiTiabdogaJnaaBaaaleaacqWGqbaucqWGVbWBcqWGSbaBaeqaaaGccaGLOaGaayzkaaGaey4kaSIaemOvay1aaSbaaSqaaiabdcfaqjabd+gaVjabdYgaSbqabaGccqGHflY1cqWGJbWydaWgaaWcbaGaemiwaGfabeaaaaa@5891@

dcGoldt=D⋅(cGolfeed−cGol)+VGol⋅cX
 MathType@MTEF@5@5@+=feaafiart1ev1aaatCvAUfKttLearuWrP9MDH5MBPbIqV92AaeXatLxBI9gBaebbnrfifHhDYfgasaacH8akY=wiFfYdH8Gipec8Eeeu0xXdbba9frFj0=OqFfea0dXdd9vqai=hGuQ8kuc9pgc9s8qqaq=dirpe0xb9q8qiLsFr0=vr0=vr0dc8meaabaqaciaacaGaaeqabaqabeGadaaakeaadaWcaaqaaiabdsgaKjabdogaJnaaBaaaleaacqWGhbWrcqWGVbWBcqWGSbaBaeqaaaGcbaGaemizaqMaemiDaqhaaiabg2da9iabdseaejabgwSixpaabmaabaGaem4yam2aa0baaSqaaiabdEeahjabd+gaVjabdYgaSbqaaiabdAgaMjabdwgaLjabdwgaLjabdsgaKbaakiabgkHiTiabdogaJnaaBaaaleaacqWGhbWrcqWGVbWBcqWGSbaBaeqaaaGccaGLOaGaayzkaaGaey4kaSIaemOvay1aaSbaaSqaaiabdEeahjabd+gaVjabdYgaSbqabaGccqGHflY1cqWGJbWydaWgaaWcbaGaemiwaGfabeaaaaa@5849@

dcEtOHdt=D⋅(cEtOHfeed−cEtOH)+VPK⋅cX
 MathType@MTEF@5@5@+=feaafiart1ev1aaatCvAUfKttLearuWrP9MDH5MBPbIqV92AaeXatLxBI9gBaebbnrfifHhDYfgasaacH8akY=wiFfYdH8Gipec8Eeeu0xXdbba9frFj0=OqFfea0dXdd9vqai=hGuQ8kuc9pgc9s8qqaq=dirpe0xb9q8qiLsFr0=vr0=vr0dc8meaabaqaciaacaGaaeqabaqabeGadaaakeaadaWcaaqaaiabdsgaKjabdogaJnaaBaaaleaacqWGfbqrcqWG0baDcqWGpbWtcqWGibasaeqaaaGcbaGaemizaqMaemiDaqhaaiabg2da9iabdseaejabgwSixpaabmaabaGaem4yam2aa0baaSqaaiabdweafjabdsha0jabd+eapjabdIeaibqaaiabdAgaMjabdwgaLjabdwgaLjabdsgaKbaakiabgkHiTiabdogaJnaaBaaaleaacqWGfbqrcqWG0baDcqWGpbWtcqWGibasaeqaaaGccaGLOaGaayzkaaGaey4kaSIaemOvay1aaSbaaSqaaiabdcfaqjabdUealbqabaGccqGHflY1cqWGJbWydaWgaaWcbaGaemiwaGfabeaaaaa@5961@
